# The effect of coenzyme Q10 supplementation on oxidative stress: A systematic review and meta‐analysis of randomized controlled clinical trials

**DOI:** 10.1002/fsn3.1492

**Published:** 2020-03-19

**Authors:** Zohreh Sadat Sangsefidi, Fatemeh Yaghoubi, Salimeh Hajiahmadi, Mahdieh Hosseinzadeh

**Affiliations:** ^1^ Nutrition and Food Security Research Center Shahid Sadoughi University of Medical Sciences Yazd Iran; ^2^ Department of Nutrition School of Public Health Shahid Sadoughi University of Medical Sciences Yazd Iran; ^3^ Department of Biochemistry Shahid Sadoughi University of Medical Sciences Yazd Iran

**Keywords:** anti‐oxidative enzymes, coenzyme Q10, malondialdehyde, meta‐analysis, oxidative stress, total antioxidant capacity

## Abstract

Some evidence exists in supporting the beneficial effects of coenzyme Q10 (CoQ10) on oxidative stress. Since the findings of studies over the impact of CoQ10 supplementation on oxidative stress are contradictory, this study was conducted. The aim was to evaluate CoQ10 supplementation effect on total antioxidant capacity (TAC), malondialdehyde (MDA), glutathione peroxidase (GPx), superoxide dismutase (SOD), and catalase (CAT) levels using data collected from randomized controlled trials (RCTs). Several databases including PubMed, Web of Science, Google Scholar, and Scopus were comprehensively searched up to 23 January 2019 to identify RCTs. A random‐effects model, standardized mean difference (SMD), and 95% confidence interval (CI) were applied for data analysis. According to the meta‐analysis results on 19 eligible studies, CoQ10 increased the levels of TAC (SMD = 1.29; 95% CI = 0.35–2.23; *p* = .007), GPX (SMD = 0.45; 95% CI = 0.17–0.74; *p* = .002), SOD (SMD = 0.63; 95% CI = 0.29–0.97; *p* < .0001), and CAT (SMD = 1.67; 95% CI = 0.29–3.10; *p* = .018) significantly. This supplementation also caused a significant reduction in MDA levels (SMD = −1.12; 95% CI = −1.58 to −0.65; *p* < .0001). However, the results of SOD and CAT should be stated carefully due to the publication bias. In conclusion, this research indicated that CoQ10 supplementation had beneficial effects on oxidative stress markers. However, further studies are needed to confirm these findings.

## Graphical abstract

This systematic review and meta‐analysis evaluated the effect of coenzyme Q10 (CoQ10) supplementation on oxidative stress markers including TAC, MDA, GPx, SOD, and CAT using data collected from RCTs. Several databases were comprehensively searched up to 23 January 2019 to identify RCTs. A random‐effects model, standardized mean difference, and 95% CI were applied for data analysis. According to the findings, CoQ10 significantly increased the levels of TAC, GPX, SOD, and CAT and in contrast decreased MDA levels.

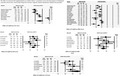

## 1 INTRODUCTION

Oxidative stress is associated with the decreased physiological activity of antioxidant defenses against free radicals. It is also defined as a disturbing factor in the balance between the production of free radicals and antioxidant defense (Sahebkar, Serban, Ursoniu, & Banach, 2015). The higher levels of oxidative stress result in increased expression of oncogenes, producing mutagenic compounds, atherogenic activity, and inflammatory processes (Kędziora‐Kornatowska et al., 2010; Pisoschi & Pop, 2015) which can increase risk of various disease such as cancer, diabetes, neurodegenerative, and cardiovascular problems (Pisoschi & Pop, 2015). Antioxidant defense system includes a wide range of factors such as coenzyme Q10 (CoQ10) and antioxidant enzymes (e.g., glutathione peroxidase [GPX], superoxide dismutase [SOD], and catalase [CAT]) (Pisoschi & Pop, 2015; Rajendran et al., 2014) which act via removing molecular oxygen or changing its local concentration; removing metal peroxidant ions; scavenging reactive oxygen species; folding initiating radicals such as hydroxyl and alkoxyl; and breaking the radical chain sequence (Martysiak‐Żurowska & Wenta, 2012).

Coenzyme Q10 (CoQ10), a vitamin‐like substance in the respiratory chain of mitochondrial membrane, plays an important role in adenosine triphosphate synthesis (Liu, Huang, Cheng, Huang, & Lin, 2015). CoQ10 is also known as ubiquinone due to its ubiquitous presence in the nature and having quinone structure (Liu et al., 2015). Human cells can synthesize this compound from the amino acid tyrosine. In addition, CoQ10 as a part of the intracellular antioxidant system protects phospholipids and membrane proteins against free radicals (Liu et al., 2015).

Although several randomized controlled trials (RCTs) investigated the effect of CoQ10 supplementation on oxidative stress, a considerable controversy exists over this subject. Some studies indicated that CoQ10 supplementation had some beneficial impacts on oxidative stress (Fakhrabadi, Ghotrom, Mozaffari‐Khosravi, Nodoushan, & Nadjarzadeh, 2014; Farhangi, Alipour, Jafarvand, & Khoshbaten, 2014; Gholnari et al., 2018; Sanoobar et al., 2013), whereas no significant effect was observed in other surveys (Abdollahzad, Aghdashi, Jafarabadi, & Alipour, 2015; Dai et al., 2011). For example, a significant reduction was reported in MDA level among patients with relapsing–remitting multiple sclerosis (Sanoobar et al., 2013) and rheumatoid arthritis (Abdollahzad et al., 2015) following the CoQ10 supplementation. However, no significant impact was found on MDA among patients with nonalcoholic fatty liver disease (NAFLD) (Farhangi et al., 2014). The results of another study among diabetic patients with neuropathic signs demonstrated a significant increase in TAC concentration after supplementation with CoQ10 (Fakhrabadi et al., 2014), while a significant reduction in TAC concentration was found among patients with NAFLD (Farhangi et al., 2014). These controversies were also reported for antioxidant enzymes such as GPx (Sanoobar et al., 2013; Yen, Chu, Lee, Lin, & Lin, 2018) and SOD (Dai et al., 2011; Lee, Huang, Chen, & Lin, 2012). Recently, a systematic review and meta‐analysis regarding effect of CoQ10 on inflammatory and some oxidative stress markers on 13 RCT among coronary artery disease (CAD) patients reported that CoQ10 supplementation was associated with increased SOD and CAT levels and decreased MDA levels (Jorat et al., 2019). However, this meta‐analysis included few trials and has evaluated CoQ10 effect only among CAD patients. Weak search strategy, linguistic limitations in search, and lack of assessing CoQ10 impact on other oxidative stress markers such as TAC are other limitations in meta‐analysis conducted by Jorat et al. (2019). Moreover, the mentioned meta‐analysis (Jorat et al., 2019) included trials with CoQ10 or CoQ10 plus other supplements that this issue might impact on its findings.

As regards, the results of literature about impact of CoQ10 supplementation on oxidative stress are controversial and the recent meta‐analysis could not accurately indicated CoQ10 effect on oxidative stress markers due to its limitations, the present systematic review and meta‐analysis was conducted. The aim of this systematic review and meta‐analysis over the published RCTs was to assess the effect of CoQ10 supplementation on oxidative stress biomarkers (including TAC, MDA, GPx, SOD, and CAT) to provide a more accurate estimate of the overall CoQ10 effect.

## 2 MATERIALS AND METHODS

### 2.1 Search strategy

The current systematic review and meta‐analysis was carried out based on the Preferred Reporting Items for Systematic Reviews and Meta‐Analyses Guidelines (PRISMA) (Moher et al., 2015). To identify eligible studies, various databases including PubMed (http://www.pubmed.com), ISI Web of Science (http://www.webofknowledge.com), Scopus (http://www.scopus.com), and Google Scholar (http://www. scholar.google.com) were searched up to 23 January 2019 without any restrictions. To hit this target, Medical Subject Heading (MeSH) terms and non‐MeSH terms were used to assess the effect of coenzyme Q10 supplementation on oxidative stress. The following keywords were applied to search:

("coenzyme Q10"[Supplementary Concept] OR "coenzyme Q10" [tiab] OR "co‐enzyme Q10" [tiab] OR ubiquinone[tiab] OR CoQ10[tiab] OR ubidecarenone[tiab]) AND ("oxidative stress"[MeSH] OR "oxidative stress"[tiab] OR "oxidative stress index"[tiab] OR OSI[tiab] OR "oxidative stress indices"[tiab] OR "oxidative stress biomarkers"[tiab] OR "oxidative stress markers"[tiab] OR "total oxidant status"[tiab] OR TOS[tiab] OR malondialdehyde[MeSH] OR malondialdehyde[tiab] OR MDA[tiab] OR "thiobarbituric acid‐reactive substances"[MeSH] OR "thiobarbituric acid‐reactive substances"[tiab] OR TBATRS[tiab] OR "total antioxidant capacity"[tiab] OR TAC[tiab] OR "total antioxidant status"[tiab] OR TAS[tiab] OR "glutathione peroxidase"[MeSH] OR "glutathione peroxidase"[tiab] OR GPx[tiab] OR "superoxide dismutase"[MeSH] OR "superoxide dismutase"[tiab] OR SOD[tiab] OR "F_2_‐isoprostanes"[MeSH] OR "F_2_‐isoprostanes"[tiab] OR catalase[MeSH] OR catalase[tiab] OR CAT[tiab]) OR glutathione[MeSH] OR glutathione[tiab] OR GSH[tiab])).

Moreover, to ensure about the comprehensiveness of searches, reference lists of the included surveys were also checked for further possible sources.

### 2.2 Selection criteria

The selected studies (a) had RCT design, (b) investigated the effect of CoQ10 supplementation on oxidative stress markers (serum or plasma), (c) reported the administered CoQ10 dosage, (d) included participants of ≥18 years, (e) did not have a duration of <28 days, and (6) presented sufficient information for oxidative stress markers (serum or plasma).

### 2.3 Study selection

**Figure 1:**
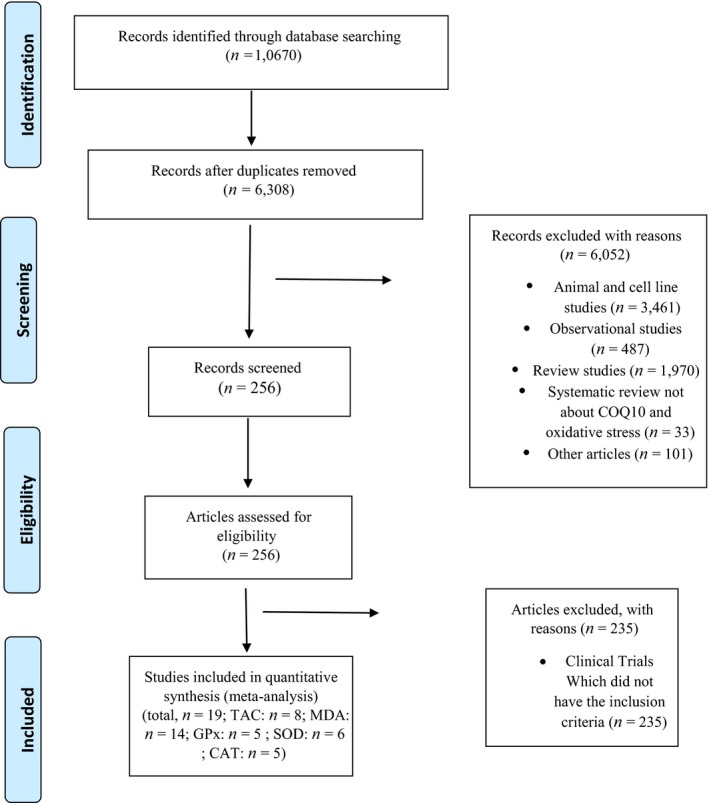
Flow chart of studies selection process

The initial screening was performed by two independent researchers (ZS.S and F.Y), who studied the articles' titles and abstracts. Then, the full texts of all related articles were assessed by reviewers to select the trials about the effect of CoQ10 supplementation on oxidative stress markers. Finally, any possible disagreement was negotiated and resolved via consultation with the third researcher (M.H) (Figure 1).

### 2.4 Data extraction

Information was extracted from the selected trials according to the following criteria: authors' family names; publication year; sample size; loss to follow‐up; dose of intervention; study duration; cross‐over or parallel study design; participants' gender, age, and health status; mean and standard deviation (*SD*) of oxidative stress markers at the beginning and at the end of the trial, as well as the mean changes and *SD* of markers' levels.

### 2.5 Quality assessment

Quality of the included studies was assessed based on the quantitative 5‐point Jadad scale (Jadad et al., 1996) by two reviewers (ZS.S and F.Y) independently. According to this scale, trials received 0–1 point based on the five sections of (a) randomization, (b) description of randomization procedure, (c) double blinding, (d) method of double blinding, and (e) description of dropouts and withdrawals. Eventually, in the case that the survey acquired ⩾3 scores, it was considered as a high‐quality study.

### 2.6 Data synthesis and analysis

Standardized mean difference (SMD) was defined as the effect size, calculated after dividing the mean by the standard deviation (*SD*) of a difference between the two random values taken from each groups (Higgins & Green, 2011). In studies that the standard error (*SE*) value was reported, *SE* was converted to *SD* using the following formula: *SD* = *SE *× √*n* (*n* = number of participants in each group). The random‐effects model and the standardized mean differences (SMDs) with 95% confidence intervals (CIs) were used for conducting the meta‐analysis (Borenstein, Hedges, Higgins, & Rothstein, 2011). Heterogeneity of trials was also evaluated using Cochran's *Q* test and was quantified by the *I*‐squared (*I* ^2^) statistic. Heterogeneity was defined as follows: *Q* statistic *p* value of <.1; weak heterogeneity: *I* ^2^ = 25–50, rather moderate heterogeneity: *I* ^2^ = 50–75, high heterogeneity: *I* ^2^ = 75–100 (Higgins & Thompson, 2002). Moreover, subgroup analysis was conducted to explore the possible sources of heterogeneity among the selected trials. Since the dose of administered COQ10, intervention duration, and study quality might have affected the findings about impact of COQ10, the subgroup analysis was conducted according to these variables. Meanwhile, publication bias was evaluated by assessing the funnel plot; mean differences were plotted against their corresponding standard errors. Moreover, formal testing was carried out for “funnel plot” asymmetry using Begg's rank correlation test and Egger's regression test at the *p* value of <.05 (Duval & Tweedie, 2000). To determine the effect of a specific trial or a particular group of trials, sensitivity analysis was carried out by individual removal of each study and recalculation of the pooled estimates. Meta‐regression was also performed to assess the relationship of the estimated effect size with CoQ10 dosage and trial duration.

Statistical analyses were performed by STATA software, version 11.2 (STATA Corp.). The statistically significant values were set at *p* < .05.

## 3 RESULTS

### 3.1 Study selection and characteristics

Our electronic search of PubMed, Web of Science, Scopus, and Google scholar resulted in 10,670 articles. After excluding duplicates, 6,308 studies remained. Of this number, 6,287 surveys were excluded since they were not clinical trials (*n* = 6,052) or did not meet the inclusion criteria (*n* = 235). Finally, 21 studies met the inclusion criteria and were investigated in our systematic review (Figure 1). Characteristics of the included studies are indicated in Tables S1–S7. All surveys were published from 1997 to 2019. The total number of participants who completed the studies in the included trials was 1,170, 614 participants in the intervention and 556 in the placebo groups. All studies had a RCT parallel design except one trial that had cross‐over design (Hamilton, Chew, & Watts, 2009). In addition, all participants were patients with various diseases such as type two diabetes mellitus (Fakhrabadi et al., 2014; Fallah, Askari, Soleimani, Feizi, & Asemi, 2019; Gholami, Zarei, Sadeghi Sedeh, Rafiei, & Khosrowbeygi, 2018; Gholnari et al., 2018; Hamilton et al., 2009; Moazen, Mazloom, Ahmadi, Dabbaghmanesh, & Roosta, 2015; Yen et al., 2018; Zarei et al., 2018), NAFLD (Farhangi et al., 2014), multiple sclerosis (Sanoobar et al., 2013), rheumatoid arthritis (Abdollahzad et al., 2015), ischemic left ventricular systolic dysfunction (Dai et al., 2011), coronary artery disease (Lee et al., 2012; Lee, Tseng, Yen, & Lin, 2013), chronic renal failure (Rivara et al., 2017; Singh, Khanna, & Niaz, 2000; Singh et al., 2003), hepatocellular carcinoma (Liu et al., 2015), metabolic syndrome (Raygan, Rezavandi, Tehrani, Farrokhian, & Asemi, 2016), and dyslipidemia (Zhang et al., 2018). However, participants of one study (Kaikkonen et al., 1997) were healthy individuals. Trial durations fluctuated from 28 to 168 days, and administered CoQ10 dosage changed from 60 to 1,200 mg/day.

### 3.2 Quality assessment of the studies

According to the Jadad criteria (Jadad et al., 1996), quality scores of the included studies (*n* = 21) ranged from 2 to 5, and all had high‐quality except four studies (Hamilton et al., 2009; Kaikkonen et al., 1997; Lee et al., 2012; Moazen et al., 2015). All the studies were randomized, but only 12 surveys (Abdollahzad et al., 2015; Dai et al., 2011; Gholnari et al., 2018; Kaikkonen et al., 1997; Lee et al., 2013; Liu et al., 2015; Moazen et al., 2015; Raygan et al., 2016; Rivara et al., 2017; Singh et al., 2003; Zarei et al., 2018; Zhang et al., 2018) explained the randomization method. Furthermore, all studies were double‐blind except five ones (Kaikkonen et al., 1997; Lee et al., 2012, 2013; Liu et al., 2015; Moazen et al., 2015). The method of blinding was explained in only nine surveys (Abdollahzad et al., 2015; Dai et al., 2011; Fakhrabadi et al., 2014; Raygan et al., 2016; Sanoobar et al., 2013; Singh et al., 2000; Singh et al., 2003; Zarei et al., 2018; Zhang et al., 2018). Furthermore, all studies described dropouts and withdrawals except five ones (Hamilton et al., 2009; Kaikkonen et al., 1997; Moazen et al., 2015; Singh et al., 2000; Singh et al., 2003). In this meta‐analysis, only articles (total; *n* = 19) associated with TAC (*n* = 8), MDA (*n* = 14), GPx (*n* = 5), SOD (*n* = 6), and CAT (*n* = 5) were included. In other words, meta‐analysis was not conducted for the studies related to F2‐isoprostanes (Hamilton et al., 2009; Rivara et al., 2017), 8‐isoprostanes (Dai et al., 2011; Gholami et al., 2018), and GSH (Fallah et al., 2019; Raygan et al., 2016) due to limited numbers of trials.

### 3.3 Effect of coenzyme Q10 supplementation on TAC levels

**Figure 2:**
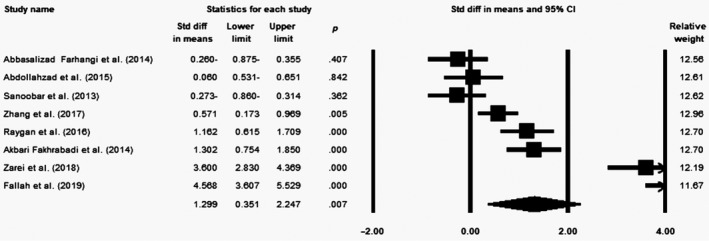
Forest plot illustrates standardized mean difference (represented by the black square) and 95% confidence interval (CI) (represented by horizontal line) for concentration of total antioxidant capacity (TAC) and coenzyme Q10 (CoQ10). Weights are from random‐effects analysis. The area of the black square is proportional to the specific study weight to the overall meta‐analysis. The center of the diamond displays the pool standardized mean differences, and its width shows the pooled 95% CI. Std diff, standard difference

Our meta‐analysis of eight eligible trials (*n* = 481, intervention: *n* = 241, placebo: *n* = 240) indicated a significant increase in TAC levels following the CoQ10 supplementation (SMD = 1.299; 95% CI = 0.351 to 2.247; *p* = .007) (Figure 2). The sensitivity analysis showed that removal of each study did not change the impact of CoQ10 on TAC (Figure S1). Moreover, the investigations indicated that the included surveys had high heterogeneity (*p* < .0001, *I* ^2^ = 95.19). According to the subgroup analysis, the effect of CoQ10 on TAC was only significant at dose >100 mg/day (SMD = 1.486; 95% CI = 0.04–2.932; *p* = .044) compared with the dose equal to 100 (SMD = 1.125; 95% CI = −0.373 to 2.623; *p* = .141) (Figure S2a). Our findings also indicated that the CoQ10 impact was significant only in supplementation duration of >60 days (SMD = 1.913; 95% CI = 0.45–3.376; *p* = .01) in comparison with ≤60 days (SMD = 0.329; 95% CI = −0.534 to 1.193; *p* = .455) (Figure S2b).

### 3.4 Effect of coenzyme Q10 supplementation on MDA levels

**Figure 3:**
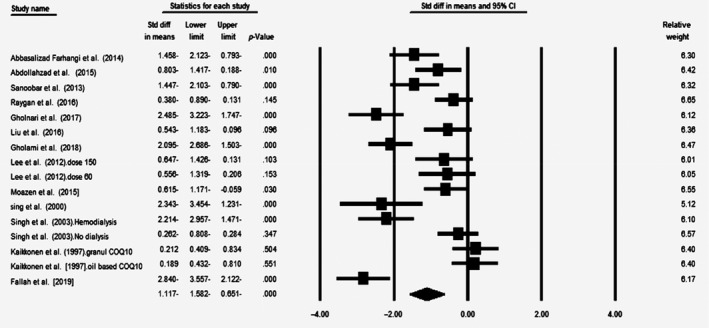
Forest plot illustrates standardized mean difference (represented by the black square) and 95% confidence interval (CI) (represented by horizontal line) for concentration of malondialdehyde (MDA) and coenzyme Q10 (CoQ10). Weights are from random‐effects analysis. The area of the black square is proportional to the specific study weight to the overall meta‐analysis. The center of the diamond displays the pool standardized mean differences, and its width shows the pooled 95% CI. Std diff, standard difference

According to meta‐analysis on 14 included surveys (*n* = 715, intervention: *n* = 361, placebo: *n* = 354), supplementation with CoQ10 decreased the MDA levels significantly (SMD = −1.117; 95% CI = −1.582 to −0.651; *p* < .0001) (Figure 3). In sensitivity analysis, the effect of CoQ10 on MDA did not change after removing each study (Figure S3). In addition, the studies had high heterogeneity (*p* < .0001, *I* ^2^ = 87.6). Subgroup analysis showed that the effect of CoQ10 on MDA was significantly higher in dosage >100 mg/day (SMD = −1.327; 95% CI = −1.996 to −0.658; *p* < .0001) in comparison with dosage ≤100 mg/day (SMD = −0.914; 95% CI = −1.594 to −0.235; *p* = .008) (Figure S4a). Furthermore, supplementation with CoQ10 for duration >60 days (SMD = −1.449; 95% CI = −2.094 to −0.804; *p* < .0001) had a significantly greater impact in comparison with duration ≤60 days (SMD = −0.663; 95% CI = −1.198 to −0.128; *p* = .015) (Figure S4b). The effect of CoQ10 was significant only among high‐quality (SMD = −1.5; 95% CI = −2.055 to −0.945; *p* < .0001) than low‐quality surveys (SMD = −0.262; 95% CI = −0.661 to 0.137; *p* = .198) (Figure S4c).

### 3.5 Effect of coenzyme Q10 supplementation on GPx levels

**Figure 4:**
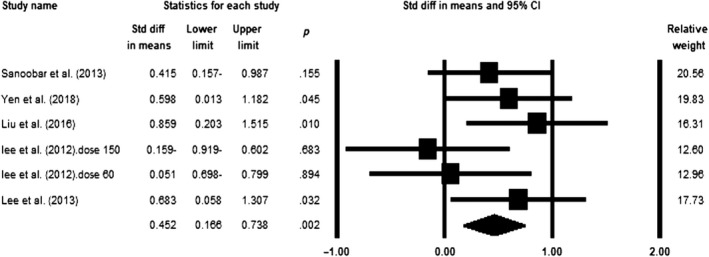
Forest plot illustrates standardized mean difference (represented by the black square) and 95% confidence interval (CI) (represented by horizontal line) for concentration of glutathione peroxidase (GPx) and coenzyme Q10 (CoQ10). Weights are from random‐effects analysis. The area of the black square is proportional to the specific study weight to the overall meta‐analysis. The center of the diamond displays the pool standardized mean differences, and its width shows the pooled 95% CI. Std diff, standard difference

Meta‐analysis of five eligible trials (*n* = 231, intervention: *n* = 122, placebo: *n* = 109) showed a significant increase in the levels of GPx following CoQ10 supplementation (SMD = 0.452; 95% CI = 0.166–0.738; *p* = .002) (Figure 4). The effect of CoQ10 did not change after removing each study in the sensitivity analysis (Figure S5). No significant heterogeneity was observed across the studies (*p* = .32, *I* ^2^ = 14.32). Subgroup analysis indicated that the impact of CoQ10 on GPx was significant only in dose >100 mg/day (SMD = 0.48; 95% CI = 0.086–0.874; *p* = .017) versus ≤100 mg/day (SMD = 0.376; 95% CI = −0.15 to 0.903; *p* = .161) (Figure S6a). Moreover, supplementation with CoQ10 demonstrated a significant effect only across high‐quality studies (SMD = 0.622; 95% CI = 0.319 to 0.926; *p* < .0001) in comparison with low‐quality articles (SMD = −0.052; 95% CI = −0.586 to 0.481; *p* = .848) (Figure S6b).

### 3.6 Effect of coenzyme Q10 supplementation on SOD levels

**Figure 5:**
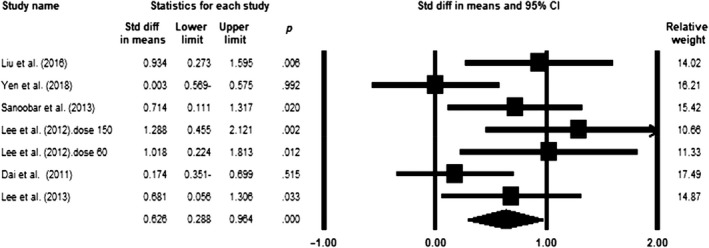
Forest plot illustrates standardized mean difference (represented by the black square) and 95% confidence interval (CI) (represented by horizontal line) for concentration of superoxide dismutase (SOD) and coenzyme Q10 (CoQ10). Weights are from random‐effects analysis. The area of the black square is proportional to the specific study weight to the overall meta‐analysis. The center of the diamond displays the pool standardized mean differences, and its width shows the pooled 95% CI. Std diff, standard difference

Based on the meta‐analysis conducted over six included studies (*n* = 284, intervention: *n* = 148, placebo: *n* = 136), supplementation with CoQ10 led to a significant increase in the levels of SOD (SMD = 0.626; 95% CI = 0.288–0.964; *p* < .0001) (Figure 5). Even after removal of each study in the sensitivity analysis, the impact of CoQ10 on SOD did not change (Figure S7). A weak heterogeneity was observed across the studies (*p* = .07, *I* ^2^ = 47.99). Based on subgroup analysis, the effect of CoQ10 on SOD was only significant in dosage >100 mg/day (SMD = 0.691; 95% CI = 0.336–1.044; *p* < .0001) versus dose ≤100 mg/day (SMD = 0.472; 95% CI = −0.521 to 1.464; *p* = .351) (Figure S8a). In addition, our findings showed that the impact of CoQ10 was significantly higher in low‐quality surveys (SMD = 1.147; 95% CI = 0.572–1.722; *p* < .0001) than the high‐quality studies (SMD = 0.473; 95% CI = 0.129–0.818; *p* = .007) (Figure S8b).

### 3.7 Effect of coenzyme Q10 supplementation on CAT levels

**Figure 6:**
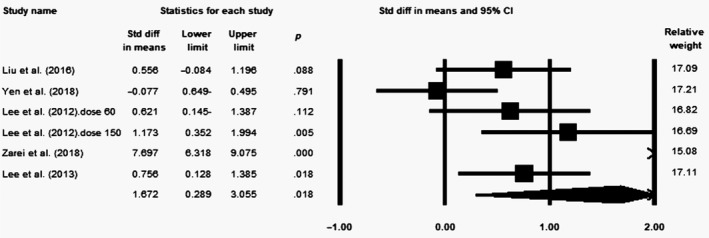
Forest plot illustrates standardized mean difference (represented by the black square) and 95% confidence interval (CI) (represented by horizontal line) for concentration of catalase (CAT) and coenzyme Q10 (CoQ10). Weights are from random‐effects analysis. The area of the black square is proportional to the specific study weight to the overall meta‐analysis. The center of the diamond displays the pool standardized mean differences, and its width shows the pooled 95% CI. Std diff, standard difference

Meta‐analysis of five eligible trials (*n* = 251, intervention: *n* = 132, placebo: *n* = 119) demonstrated a significant increase in CAT levels following the supplementation with CoQ10 (SMD = 1.672; 95% CI = 0.289–3.055; *p* = .018) (Figure 6). In sensitivity analysis, the effect of CoQ10 did not change after removal of each study (Figure S9). Moreover, high heterogeneity was observed among the studies (*p* < .0001, *I* ^2^ = 95.31). Subgroup analysis showed that the effect of CoQ10 on CAT was significant only in supplementation doses of >100 mg/day (SMD = 0.776; 95% CI = 0.383–1.17; *p* < .0001) compared with doses of ≤100 mg/day (WMD = 2.681; 95% CI = −0.796 to 6.158; *p* = .131) (Figure S10a). The effect of CoQ10 was also significantly higher across high‐quality studies (SMD = 2.121; 95% CI = 0.005–4.237; *p* = .049) than the low‐quality articles (SMD = 0.878; 95% CI = 0.318–1.438; *p* = .002) (Figure S10b).

### 3.8 Meta‐regression

Meta‐regression analysis was carried out in order to evaluate the relationship of changes in the levels of oxidative stress markers with different administered CoQ10 doses and duration of supplementation. Although a significant association was observed between CoQ10 dosage and TAC level (slope = −0.003; 95% CI = −0.004 to −0.001; *p* = .0002) (Figure S11a), no significant relationship was found between the dose of CoQ10 and other oxidative stress markers' concentrations (MDA: slope = −0.0007, 95% CI = −0.002 to 0.0008, *p* = .36; GPx: slope = 0.0006, 95% CI = −0.001 to 0.002, *p* = .45; SOD: slope = 0.0003, 95% CI = −0.001 to 0.002, *p* = .72; CAT: slope = −0.001, 95% CI = −0.004 to 0.001, *p* = .41) (Figure S11b,c,d,e, respectively). Our results showed a significant relationship between the supplementation duration and the levels of MDA (Slope: −0.011, 95% CI = −0.021 to −0.002, *p* = .01), whereas no significant association was found regarding TAC (Slope = 0.0005, 95% CI = −0.003 to 0.004, *p* = .78) (Figure S12a,b, respectively).

### 3.9 Publication bias

Based on the funnel plots and asymmetry tests, publication bias was not confirmed for studies related to TAC (Begg's test *p* = .38 and Egger'test *p* = .0.09), MDA (Begg's test *p* = .07 and Egger'test *p* = .053), and GPx (Begg's test *p* = .7 and Egger'test *p* = .15) (Figure S13a,b,c). However, funnel plots and asymmetry tests indicated a significant publication bias for surveys related to SOD (Begg's test *p* = .01 and Egger'test *p* = .01) (Figure S13d). After adjusting the effect size for potential publication bias using the “trim and fill” correction, two potentially missing surveys were imputed in funnel plot (WMD = 0.479, 95% CI = 0.143–0.814) (Figure S14a). For trials associated with CAT, funnel plots and Begg's test did not show any significant publication bias (Begg's test *p* = .06) (Figure S13e). Nevertheless, Egger's test represented a significant publication bias (Egger'test *p* = .005). After adjusting the effect size for potential publication bias by the “trim and fill” correction, no potentially missing surveys were needed in the funnel plot (WMD = 1.671, 95% CI = 0.289–3.055) (Figure S14b).

## 4 DISCUSSION

The findings of this study showed that CoQ10 supplementation increased the levels of TAC and antioxidant enzymes (including SOD, CAT, and GPx) significantly. However, it decreased the MDA levels significantly. Nevertheless, the results of SOD and CAT should be stated carefully due to the publication bias. Significant associations were also observed between the dose of this supplement and TAC levels and as well as between the supplementation period and MDA. However, no significant relationship was found between CoQ10 dose, the levels of antioxidant enzymes (including GPx, SOD, and CAT), and MDA.

Several systematic reviews and meta‐analyses showed the protective effects of this supplement on the inflammatory markers (Fan et al., 2017; Mazidi, Kengne, Banach, Lipid, & Group, 2018; Zhai, Bo, Lu, Liu, & Zhang, 2017), glycemic indices (Stojanović & Radenković, 2017; Suksomboon, Poolsup, & Juanak, 2015), lipid profiles (Jorat et al., 2018; Sahebkar, Simental‐Mendía, Stefanutti, & Pirro, 2016), and blood pressure (Rosenfeldt et al., 2007). Similar to our findings, these studies attributed the beneficial effects of CoQ10 to its antioxidant effect. Recently, a systematic review and meta‐analysis about effect of CoQ10 on inflammatory and oxidative stress (SOD, CAT, MDA, GPx, and diene) on 13 trials among coronary artery disease patients (CAD) indicated that supplementation with CoQ10 resulted in increased SOD and CAT levels and decreased MDA levels, while no significant impact of CoQ10 was found on GPx concentration (Jorat et al., 2019). Nevertheless, this meta‐analysis (Jorat et al., 2019) included fewer studies (13 trials) in comparison with our research (19 surveys). The effect of CoQ10 on TAC level as an oxidative stress marker was not evaluated in meta‐analysis conducted by Jorat et al. (2019), whereas we assess CoQ10 impact on this marker in the present meta‐analysis. Other strengths of our meta‐analysis in compared with research of Jorat et al. (2019) are stronger and more accurate search strategy and lack of linguistic limitations in search. Trials that their intervention was only CoQ10 were entered in the presented meta‐analysis, while meta‐analysis of Jorat et al. (2019) included studies with CoQ10 or CoQ10 plus other supplements. Therefore, this issue might influence on the results of study of Jorat et al. (2019). Furthermore, in our study, we assessed the effect of CoQ10 among eligible surveys with different participants including healthy subjects and patients with various diseases. However, Jorat et al. (2019) studied CoQ10 impact only among CAD patients. Meanwhile, subgroup analysis according to study quality and meta‐regression analysis based on dose and duration of administered CoQ10 were performed in the present meta‐analysis in comparison with meta‐analysis of Jorat et al. (2019).

Similarly, another meta‐analysis was conducted over the effects of CoQ10 supplementation on the metabolic profile including LDL, FBS, HDL, TG, HOMA‐IR, MDA, CRP, and creatinine in patients with chronic renal failure. In this research, seven clinical trials were investigated and the findings showed that COQ10 reduced the MDA concentration significantly (Bakhshayeshkaram et al., 2018).

The conducted clinical trials reported different results regarding the effectiveness of CoQ10 supplementation on oxidative stress. However, similar to our meta‐analysis, these studies concluded that receiving CoQ10 supplementation resulted in a significant decrease in MDA levels among patients with remitting multiple sclerosis (Sanoobar et al., 2013) and rheumatoid arthritis (Abdollahzad et al., 2015). Meanwhile, it increased TAC levels in diabetic neuropathy patients (Fakhrabadi et al., 2014). Contrary to the present study, CoQ10 did not have any significant effect on MDA and TAC in patients with NAFLD (Farhangi et al., 2014) and rheumatoid arthritis (Abdollahzad et al., 2015), respectively. In addition, similar to our research, results of some studies among patients with coronary artery disease (Lee et al., 2012) and remitting multiple sclerosis (Sanoobar et al., 2013) showed that receiving CoQ10 increased the activity of antioxidant enzymes significantly. However, other researchers did not report any significant effect of CoQ10 on the activity of antioxidant enzymes in patients with diabetes (Yen et al., 2018) or ischemic left ventricular systolic dysfunction (Dai et al., 2011).

The findings of some studies indicated that the effects of antioxidant supplements, including CoQ10 vary according to the dose, duration of use, and formulation type of the supplement in subjects with different health conditions (Bhagavan & Chopra, 2006; Bjelakovic, Nikolova, Gluud, Simonetti, & Gluud, 2007; Jankowski, Korzeniowska, Cieślewicz, & Jabłecka, 2016; Lyon et al., 2001; Poljsak, Šuput, & Milisav, 2013; Singh et al., 2005). Therefore, a possible explanation to justify these controversies can be attributed to the differences in the subjects' health status, initial levels of oxidative stress indices, dosage of CoQ10, supplementation period, and sample size of studies.

The antioxidant capacity of CoQ10 has an important role in reducing the production of free radicals, which can ultimately lead to a reduction in MDA levels (Bentinger, Brismar, & Dallner, 2007). Furthermore, antioxidant enzymes such as SOD, GPx, and CAT are responsible for neutralizing the free radicals. The activity of these enzymes increases in body followed by the consumption of antioxidants, such as CoQ10 (Limón‐Pacheco & Gonsebatt, 2009). However, the exact mechanism through which CoQ10 increases the activity of these enzymes is not completely clear. A possible mechanism is that the antioxidant compounds such as CoQ10 have a protective effect on antioxidant enzymes by absorbing free radicals and improve their activity (Limón‐Pacheco & Gonsebatt, 2009). Evidence also demonstrated that CoQ10 increased gene expression of antioxidant enzymes (Jang et al., 2017; Jorat et al., 2019). In addition, CoQ10 reduces the production of free radicals using its antioxidant capacity, oxidative stress reduction, and consequently improves the TAC levels (Mancini et al., 2004; Raygan et al., 2016).

Application of robust search strategy and study design; different subgroup analysis based on dose supplementation, trial duration, study quality, and lack of linguistic limitations in search were among the strengths of this study. However, the current meta‐analysis had several limitations. First, information was not available considering the formulation of CoQ10 supplementation used in clinical trials, since different pharmacokinetic properties may affect the bioavailability of various formulations and consequently the effects of CoQ10. No information has been presented with regard to the CoQ10 food sources and interactions of CoQ10 supplement with these food sources. The significant heterogeneity within the studied factors, except GPx, may be due to various study durations (28–168 days), supplemental doses (60–1,200 mg/day), patients' initial antioxidant serum levels, participants' health status (healthy subjects or patients with different disease), and patients' other characteristics, such as gender and age. Moreover, the clinical trials included in this meta‐analysis had limited sample sizes and follow‐up periods. Meanwhile, due to the publication bias observed in SOD‐ and CAT‐related studies, their results should be considered with cautious. As a result, the current meta‐analysis showed that CoQ10 supplementation significantly increased the levels of TAC and antioxidant enzymes (SOD, GPx and CAT) and reduced malondialdehyde levels. More clinical trials are required using stronger designs and bigger sample sizes to confirm the positive effects of CoQ10 supplementation on oxidative stress at different doses and in longer duration.

## CONFLICT OF INTEREST

The authors declare no conflict of interest to report regarding this study.

## ETHICAL STATEMENT

Not applicable.

## Supporting information

Fig S1

Fig S2

Fig S3

Fig S4

Fig S5

Fig S6

Fig S7

Fig S8

Fig S9

Fig S10

Fig S11

Fig S12

Fig S13

Fig S14

Table S1

Table S2

Table S3

Table S4

Table S5

Table S6

Table S7
