# Supplementary figures and images for: The effect of coenzyme Q10 supplementation on oxidative stress: A systematic review and meta‐analysis of randomized controlled clinical trials
Source: Food Sci Nutr. 2020 Mar 19;8(4):1766–76. doi: 10.1002/fsn3.1492 (PMC7174219; doi:10.1002/fsn3.1492)

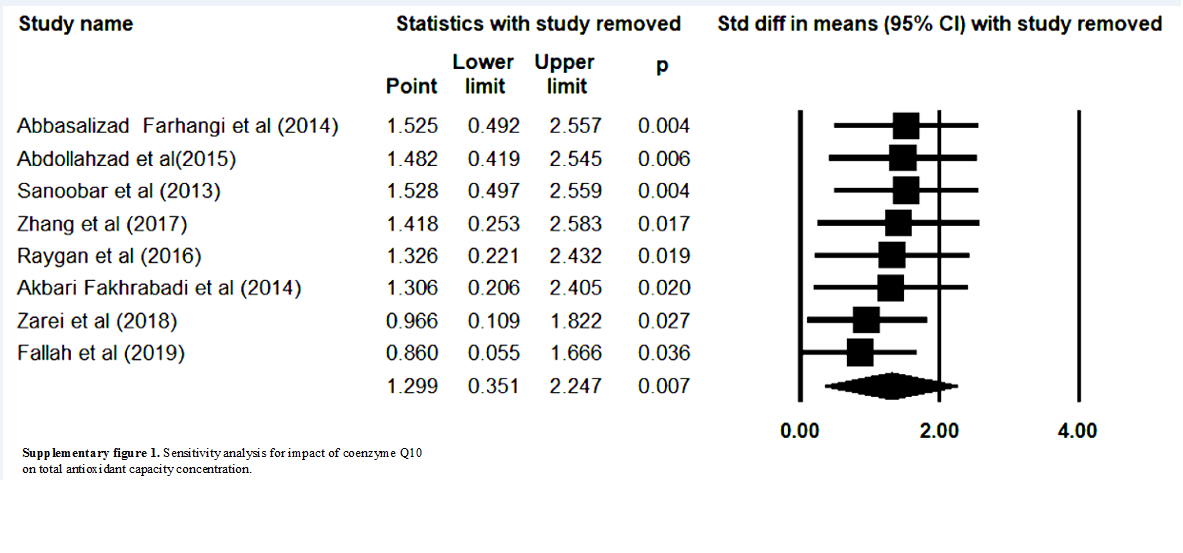

Supplement: Supplementary file 1 — Fig S1 [file FSN3-8-1766-s001.tif]

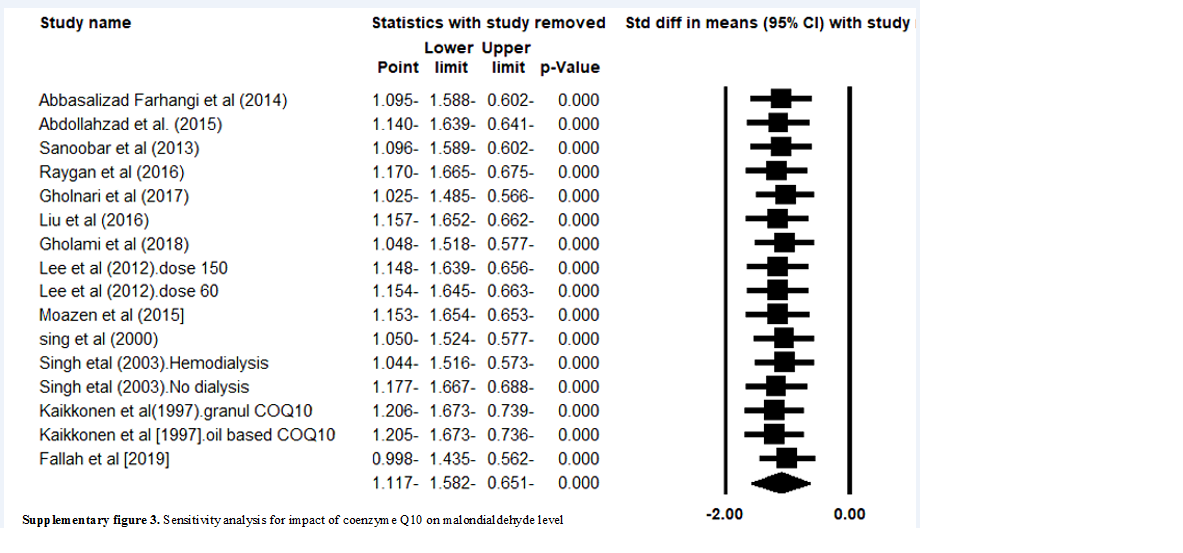

Supplement: Supplementary file 3 — Fig S3 [file FSN3-8-1766-s003.tif]

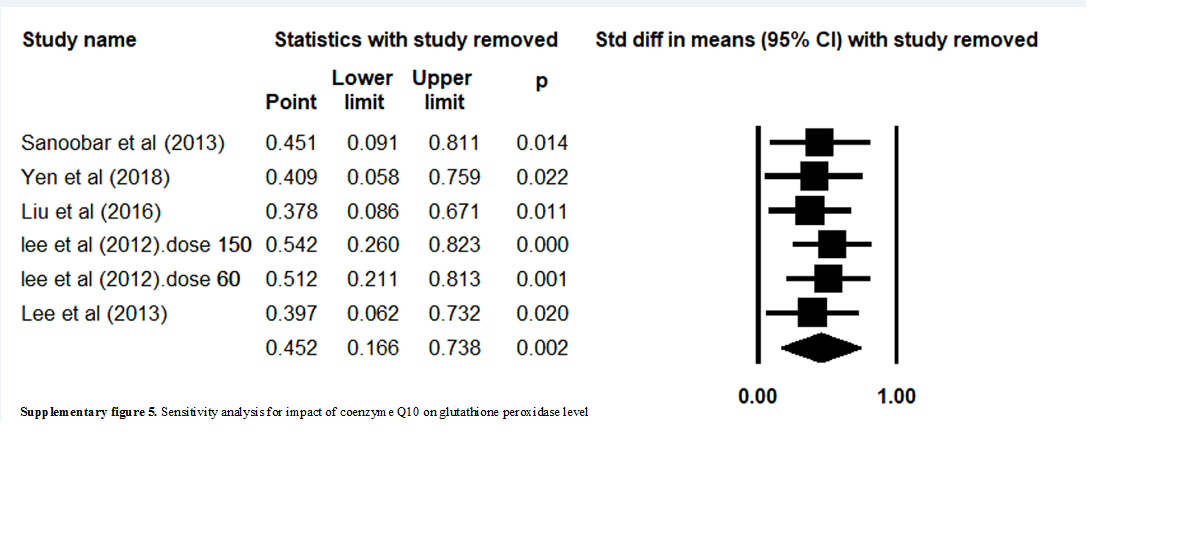

Supplement: Supplementary file 5 — Fig S5 [file FSN3-8-1766-s005.tif]

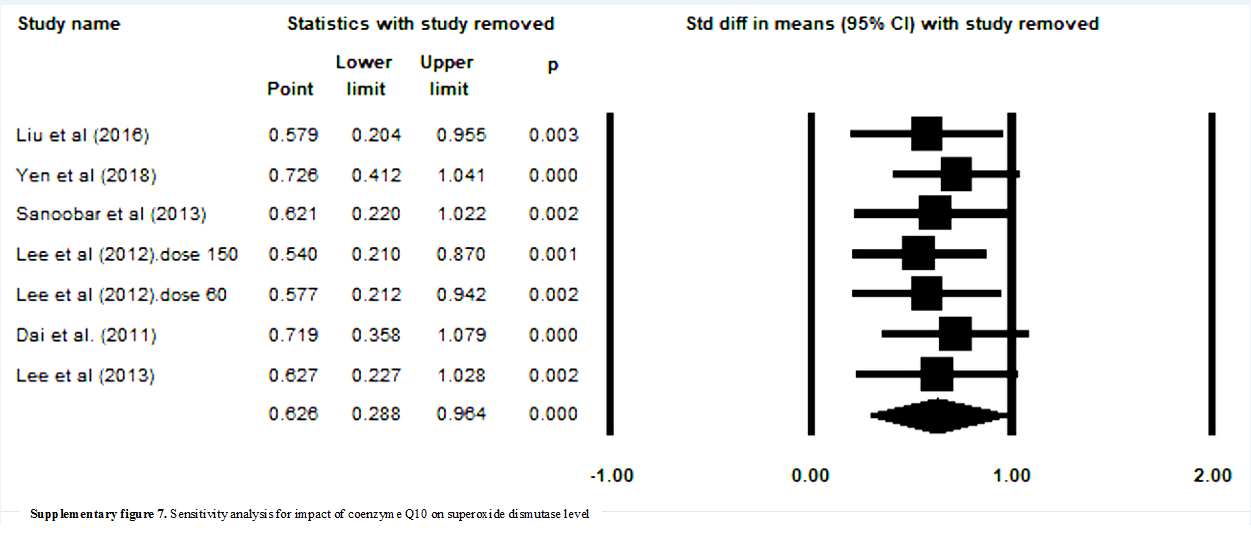

Supplement: Supplementary file 7 — Fig S7 [file FSN3-8-1766-s007.tif]

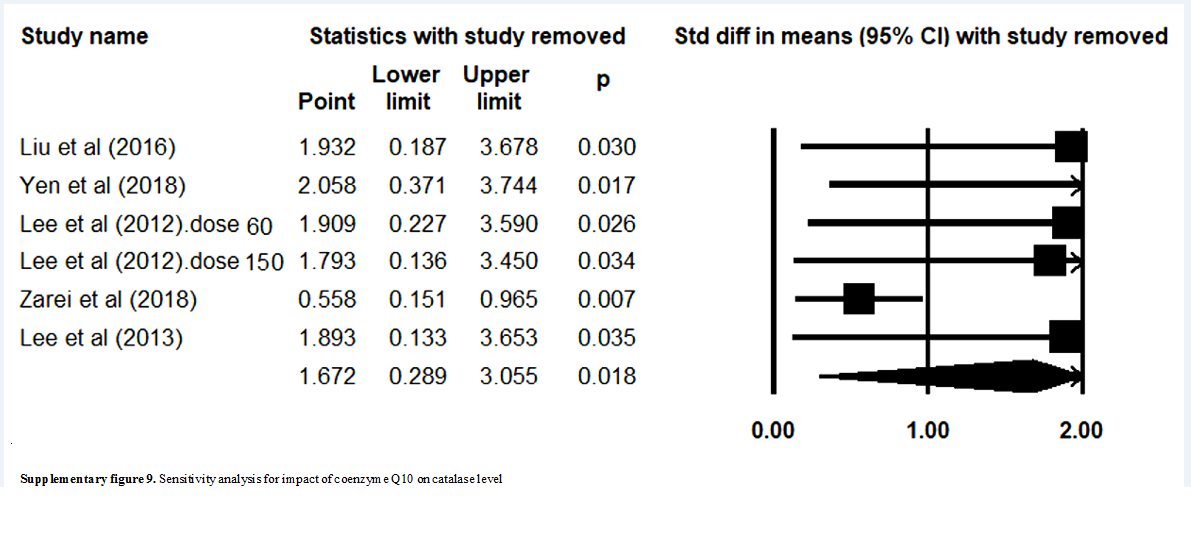

Supplement: Supplementary file 9 — Fig S9 [file FSN3-8-1766-s009.tif]

A

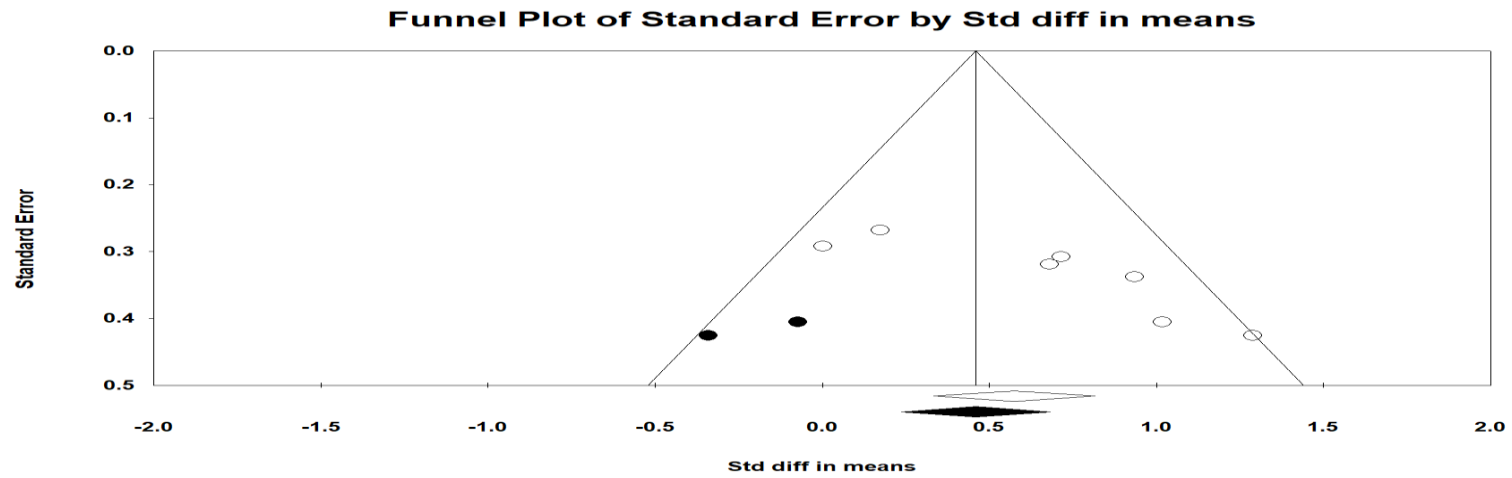

B

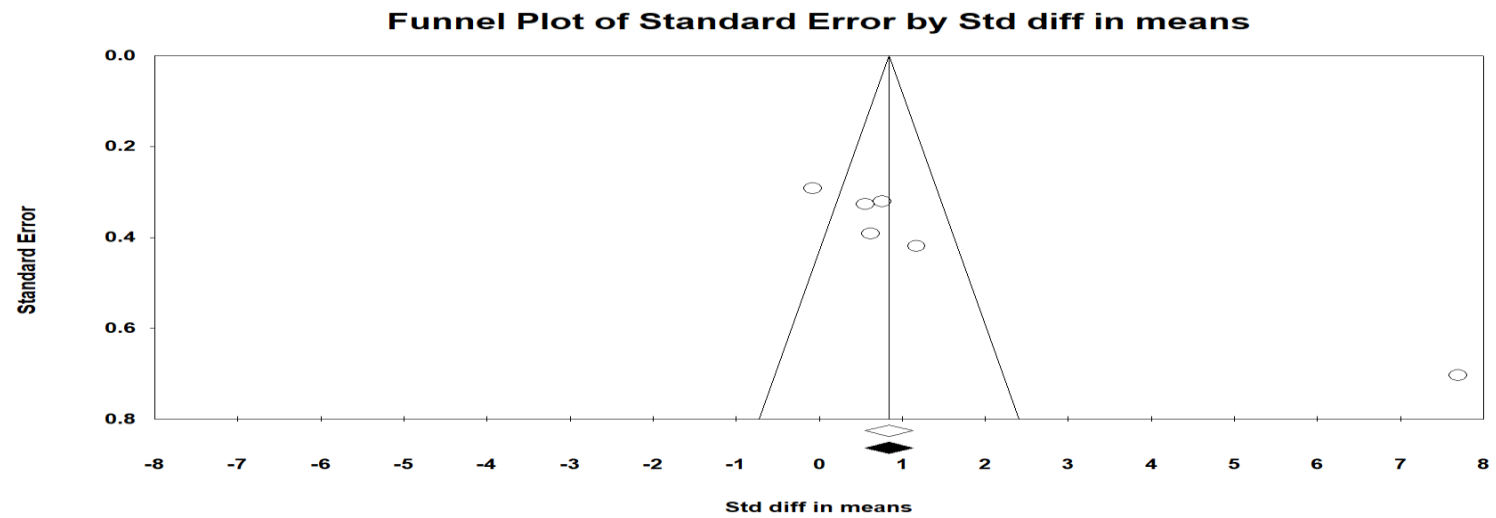

**Supplementary Figure 14.** Trim and fill method for superoxidase dismutase (A) and catalase (B).

Supplement: Supplementary file 14 — Fig S14 [file FSN3-8-1766-s014.pdf]
