# Supplementary material for: The effect of coenzyme Q10 supplementation on oxidative stress: A systematic review and meta‐analysis of randomized controlled clinical trials
Source: Food Sci Nutr. 2020 Mar 19;8(4):1766–76. doi: 10.1002/fsn3.1492 (PMC7174219; doi:10.1002/fsn3.1492)
Supplement: Supplementary file 2 — Fig S2 [file FSN3-8-1766-s002.pdf]

A

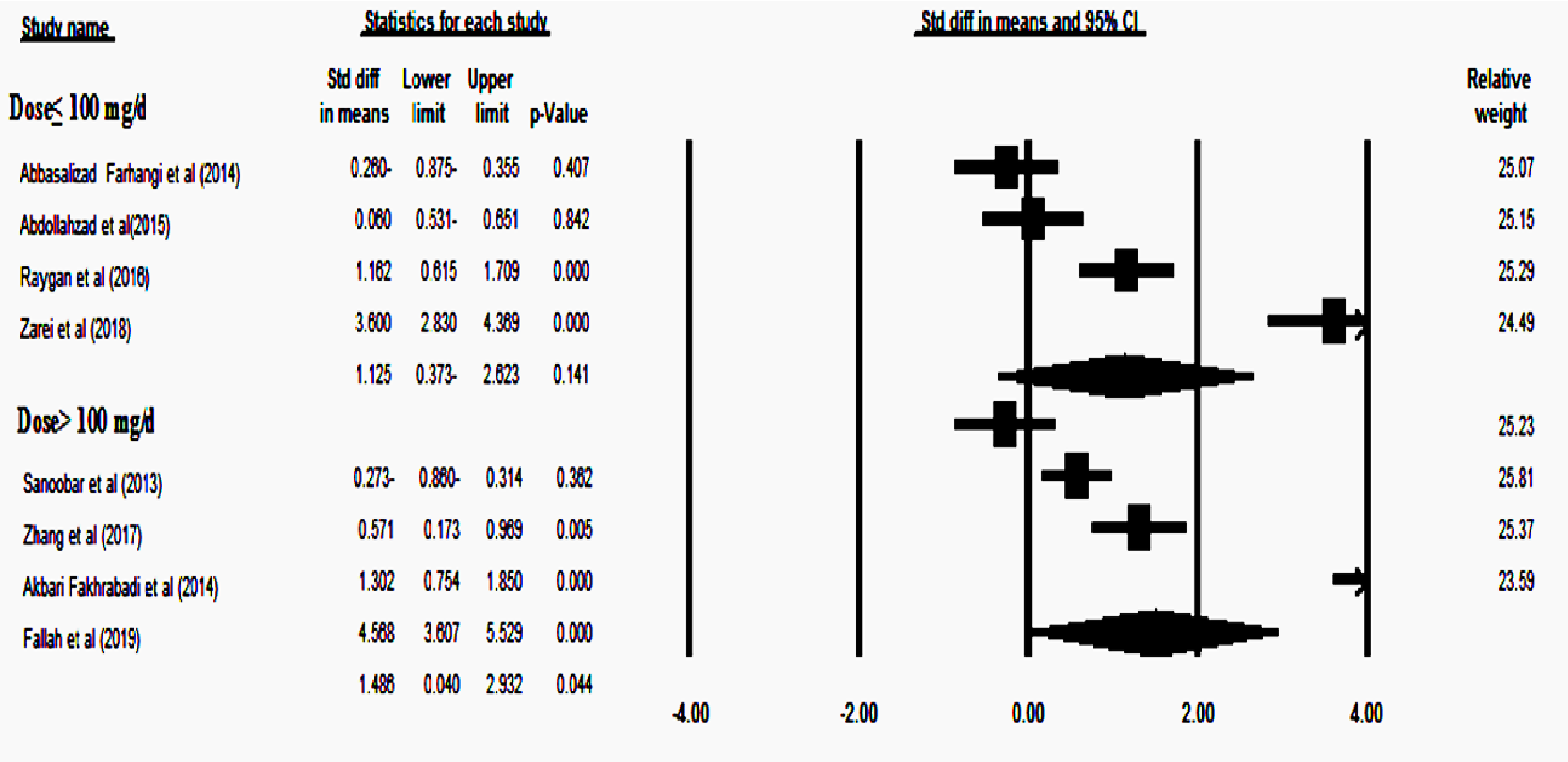

Study name
Statistics for each study
Std diff in means and 95% CI
Std diff in means
Lower limit
Upper limit
p-Value
Relative weight
Dose≤ 100 mg/d
Abbasalizad Farhangi et al (2014) 0.280- 0.875- 0.355 0.407 25.07
Abdollahzad et al(2015) 0.060 0.531- 0.651 0.842 25.15
Raygan et al (2016) 1.162 0.615 1.709 0.000 25.29
Zarei et al (2018) 3.600 2.830 4.369 0.000 24.49
1.125 0.373- 2.623 0.141
Dose> 100 mg/d
25.23
Sanoobar et al (2013) 0.273- 0.860- 0.314 0.362 25.81
Zhang et al (2017) 0.571 0.173 0.969 0.005 25.37
Akbari Fakhrabadi et al (2014) 1.302 0.754 1.850 0.000 23.59
Fallah et al (2019) 4.568 3.607 5.529 0.000
1.486 0.040 2.932 0.044
-4.00
-2.00
0.00
2.00
4.00

B

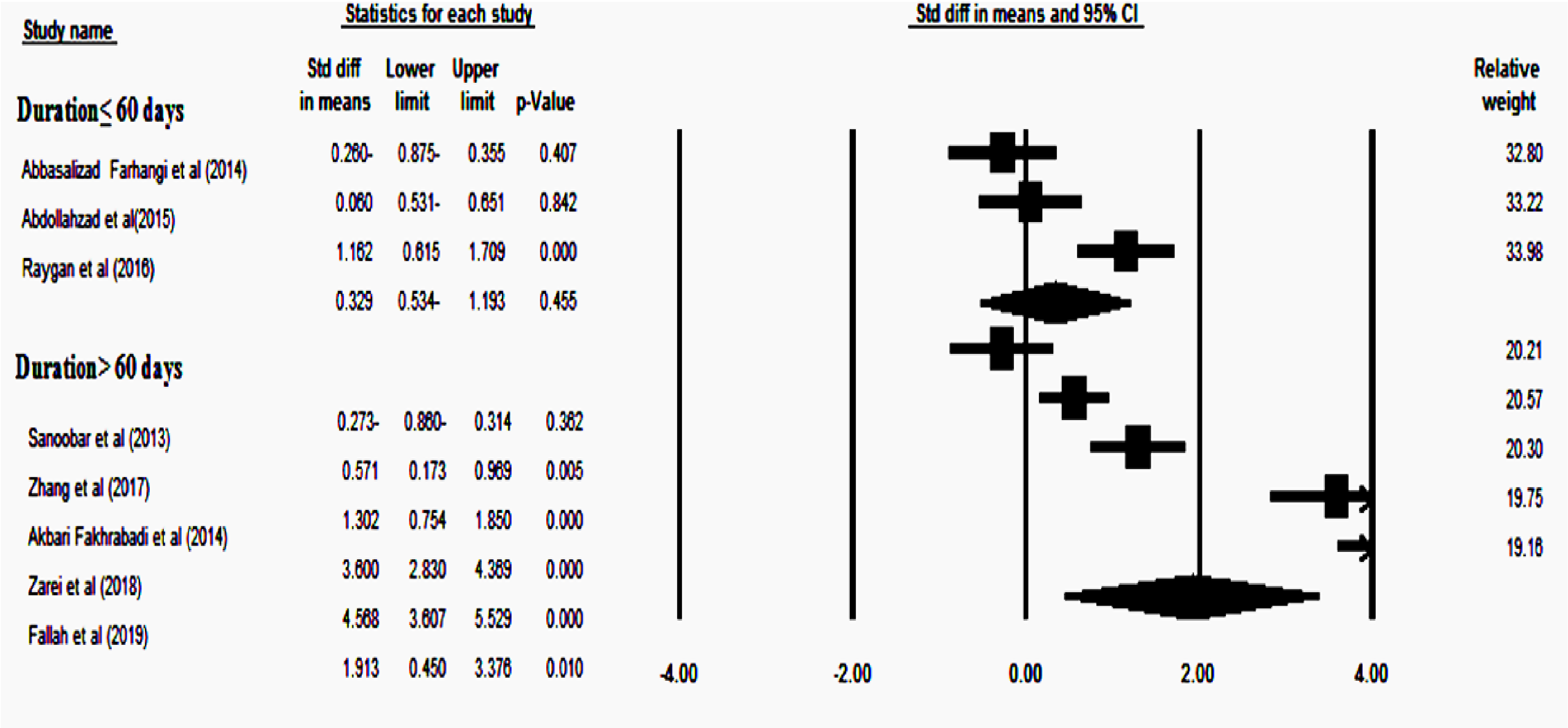

Supplementary figure 2. Subgroup analysis for effect of coenzyme Q10 (CoQ10) on total antioxidant capacity (TAC) levels based on different doses (A. dose= 100 or > 100 mg/d) and intervention durations (B. duration ≤ 60 or > 60 days).
