# Supplementary material for: The effect of coenzyme Q10 supplementation on oxidative stress: A systematic review and meta‐analysis of randomized controlled clinical trials
Source: Food Sci Nutr. 2020 Mar 19;8(4):1766–76. doi: 10.1002/fsn3.1492 (PMC7174219; doi:10.1002/fsn3.1492)
Supplement: Supplementary file 4 — Fig S4 [file FSN3-8-1766-s004.pdf]

A

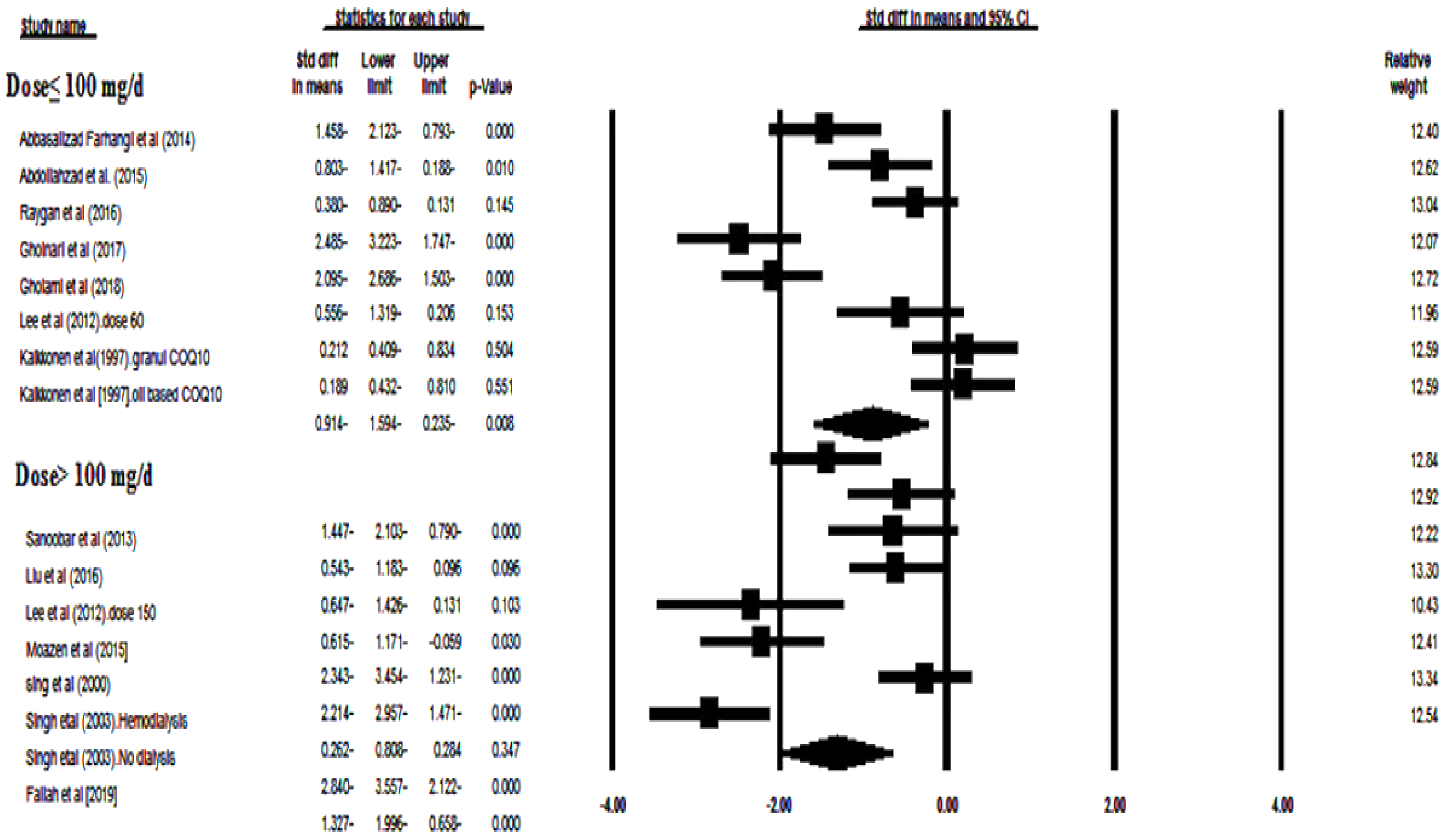

Study name
Statistics for each study
Std diff in means and 95% CI
Std diff in means
Lower limit
Upper limit
p-Value
Relative weight
Dose≤ 100 mg/d
Abbasalizad Farhangi et al (2014) 1.458- 2.123- 0.793- 0.000 12.40
Abdollahzad et al. (2015) 0.803- 1.417- 0.188- 0.010 12.62
Raygan et al (2016) 0.380- 0.890- 0.131 0.145 13.04
Gholnari et al (2017) 2.485- 3.223- 1.747- 0.000 12.07
Gholami et al (2018) 2.096- 2.686- 1.503- 0.000 12.72
Lee et al (2012).dose 60 0.556- 1.319- 0.206 0.153 11.96
Kaikkonen et al(1997).granul COQ10 0.212 0.409- 0.834 0.504 12.59
Kaikkonen et al [1997].oil based COQ10 0.189 0.432- 0.810 0.551 12.59
0.914- 1.594- 0.235- 0.008
Dose> 100 mg/d
12.84
12.92
Sanoobar et al (2013) 1.447- 2.103- 0.790- 0.000 12.22
Liu et al (2016) 0.543- 1.183- 0.096 0.096 13.30
Lee et al (2012).dose 150 0.647- 1.426- 0.131 0.103 10.43
Moazen et al (2015) 0.615- 1.171- -0.059 0.030 12.41
sing et al (2000) 2.343- 3.454- 1.231- 0.000 13.34
Singh etal (2003).Hemodialysis 2.214- 2.957- 1.471- 0.000 12.54
Singh etal (2003).No dialysis 0.262- 0.808- 0.284 0.347
Fallah et al [2019] 2.840- 3.557- 2.122- 0.000
1.327- 1.996- 0.658- 0.000
-4.00
-2.00
0.00
2.00
4.00

B

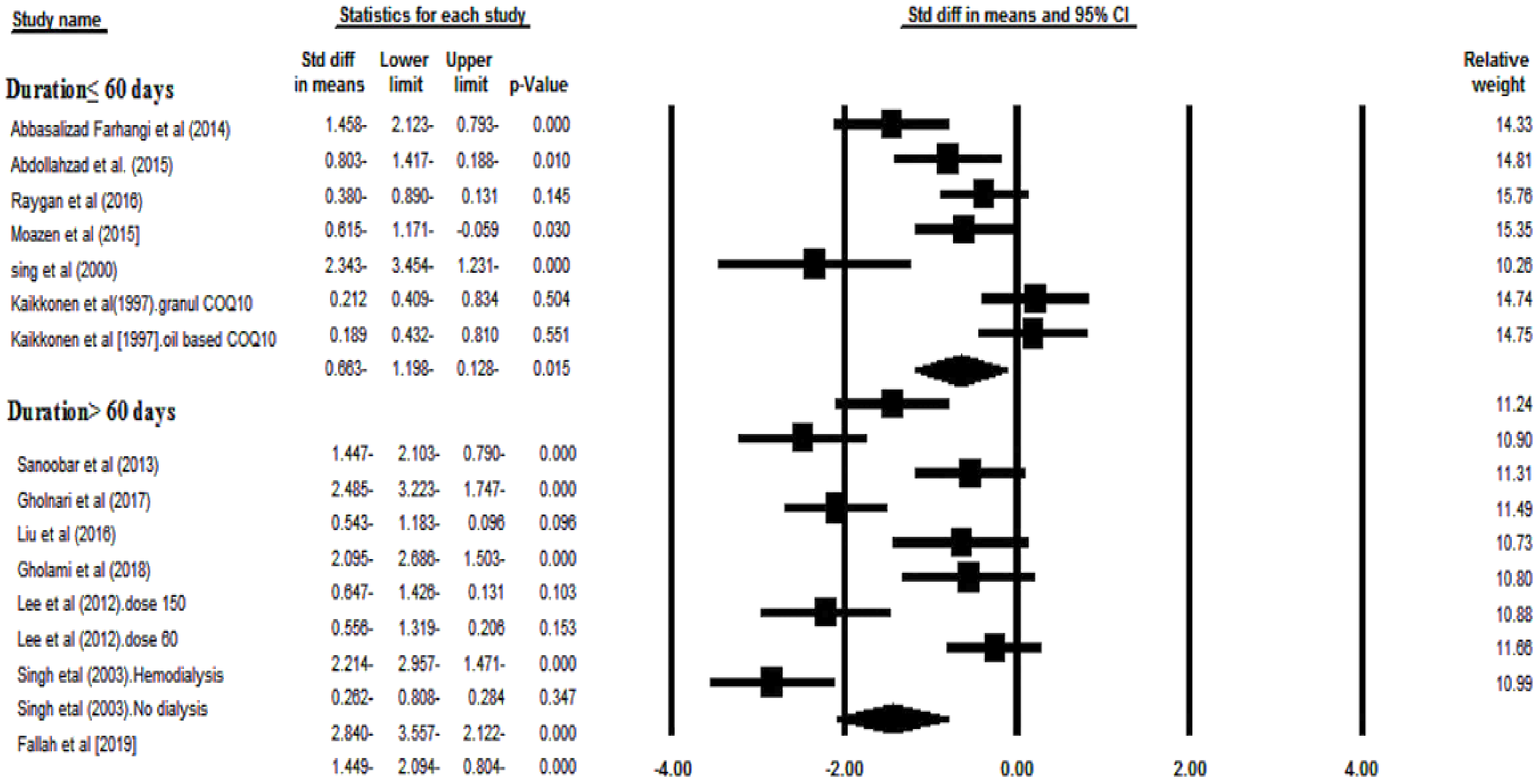

Study name
Statistics for each study
Std diff in means and 95% CI
Std diff in means
Lower limit
Upper limit
p-Value
Relative weight
Duration≤ 60 days
Abbasalizad Farhangi et al (2014) 1.458- 2.123- 0.793- 0.000 14.33
Abdollahzad et al. (2015) 0.803- 1.417- 0.188- 0.010 14.81
Raygan et al (2016) 0.380- 0.890- 0.131 0.145 15.76
Moazen et al (2015] 0.615- 1.171- -0.059 0.030 15.35
sing et al (2000) 2.343- 3.454- 1.231- 0.000 10.28
Kaikkonen et al(1997).granul COQ10 0.212 0.409- 0.834 0.504 14.74
Kaikkonen et al [1997].oil based COQ10 0.189 0.432- 0.810 0.551 14.75
0.663- 1.198- 0.128- 0.015
Duration> 60 days
Sanoobar et al (2013) 1.447- 2.103- 0.790- 0.000 11.24
Gholnari et al (2017) 2.485- 3.223- 1.747- 0.000 10.90
Liu et al (2016) 0.543- 1.183- 0.096 0.096 11.31
Gholami et al (2018) 2.095- 2.688- 1.503- 0.000 11.49
Lee et al (2012).dose 150 0.647- 1.426- 0.131 0.103 10.73
Lee et al (2012).dose 60 0.556- 1.319- 0.206 0.153 10.80
Singh etal (2003).Hemodialysis 2.214- 2.957- 1.471- 0.000 10.88
Singh etal (2003).No dialysis 0.262- 0.808- 0.284 0.347 11.68
Fallah et al [2019] 2.840- 3.557- 2.122- 0.000 10.99
1.449- 2.094- 0.804- 0.000
-4.00
-2.00
0.00
2.00
4.00

C

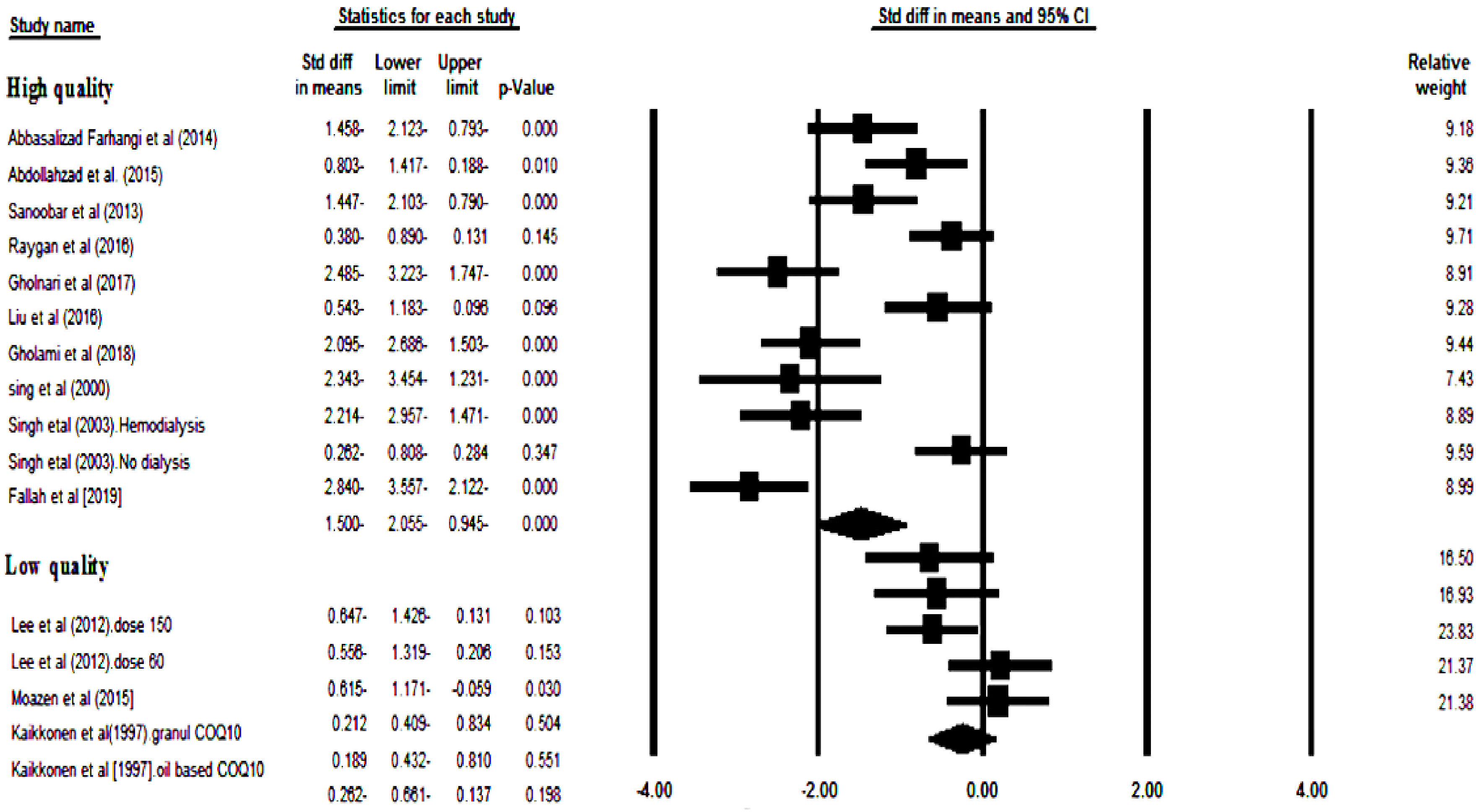

Supplementary figure 4. Subgroup analysis for effect of coenzyme Q10 (COQ10) on malondialdehyde (MDA) levels based on different doses (A. dose ≤ 100 or > 100 mg/d), intervention durations (B. duration ≤ 60 or > 60 days) and studies with different qualities (C. high quality or low quality).
