# Supplementary material for: The effect of coenzyme Q10 supplementation on oxidative stress: A systematic review and meta‐analysis of randomized controlled clinical trials
Source: Food Sci Nutr. 2020 Mar 19;8(4):1766–76. doi: 10.1002/fsn3.1492 (PMC7174219; doi:10.1002/fsn3.1492)
Supplement: Supplementary file 6 — Fig S6 [file FSN3-8-1766-s006.pdf]

A

Study name	Std diff in means	Lower limit	Upper limit	p-Value	Relative weight
**Dose≤ 100 mg/d**					
Yen et al (2018)	0.598	0.013	1.182	0.045	59.51
lee et al (2012).dose 60	0.051	0.698-	0.799	0.894	40.49
	0.376	0.150-	0.903	0.161	
**Dose> 100 mg/d**					29.35
					24.54
Sanoobar et al (2013)	0.415	0.157-	0.987	0.155	
					19.90
Liu et al (2016)	0.859	0.203	1.515	0.010	
					28.20
lee et al (2012).dose 150	0.159-	0.919-	0.602	0.683	
Lee et al (2013)	0.683	0.058	1.307	0.032	
	0.480	0.086	0.874	0.017	

Std diff in means and 95% CI

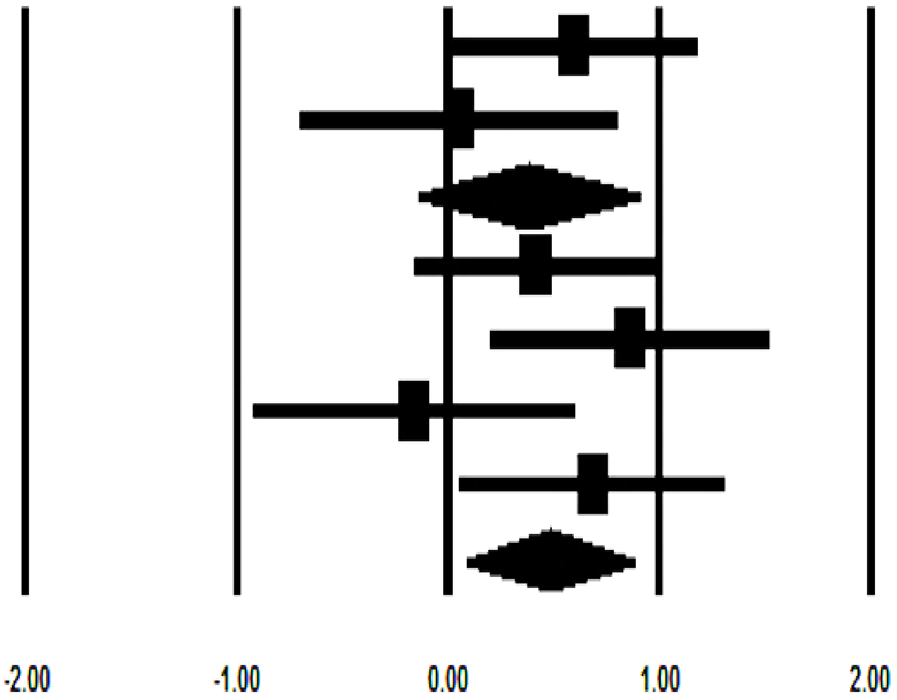

B

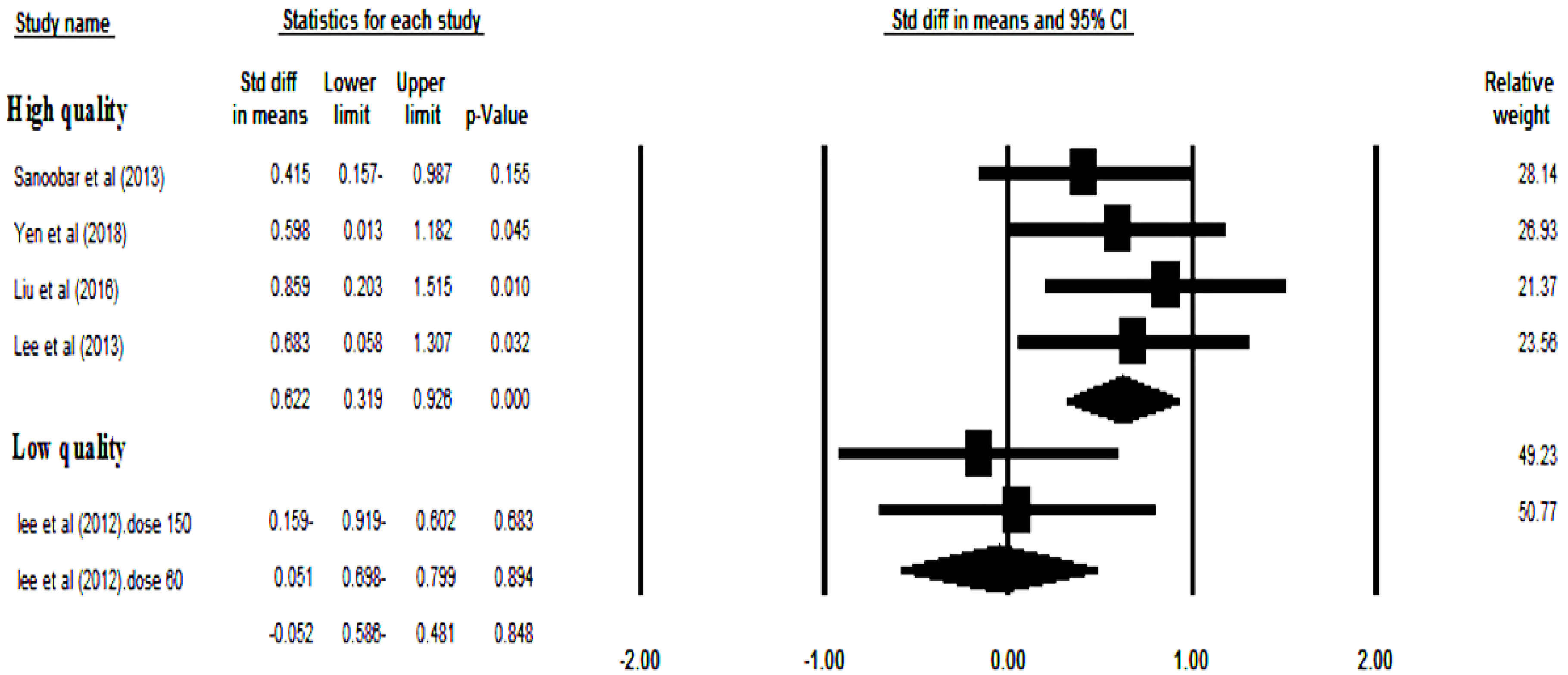

Supplementary figure 6. Subgroup analysis for effect of coenzyme Q10 (CoQ10) on glutathione peroxidase (GPx) based on different doses (A. dose ≤ 100 or > 100 mg/d) and studies with different qualities (B. high quality or low quality)
