# Supplementary material for: The effect of coenzyme Q10 supplementation on oxidative stress: A systematic review and meta‐analysis of randomized controlled clinical trials
Source: Food Sci Nutr. 2020 Mar 19;8(4):1766–76. doi: 10.1002/fsn3.1492 (PMC7174219; doi:10.1002/fsn3.1492)
Supplement: Supplementary file 8 — Fig S8 [file FSN3-8-1766-s008.pdf]

A

Study name	Std diff in means	Lower limit	Upper limit	p-Value
**Dose≤ 100 mg/d**				
Yen et al (2018)	0.003	0.569-	0.575	0.992
Lee et al (2012).dose 60	1.018	0.224	1.813	0.012
	0.472	0.521-	1.464	0.351
**Dose> 100 mg/d**				
Liu et al (2016)	0.934	0.273	1.595	0.006
Sanoobar et al (2013)	0.714	0.111	1.317	0.020
Lee et al (2012).dose 150	1.288	0.455	2.121	0.002
Dai et al. (2011)	0.174	0.351-	0.699	0.515
Lee et al (2013)	0.681	0.056	1.306	0.033
	0.691	0.338	1.044	0.000

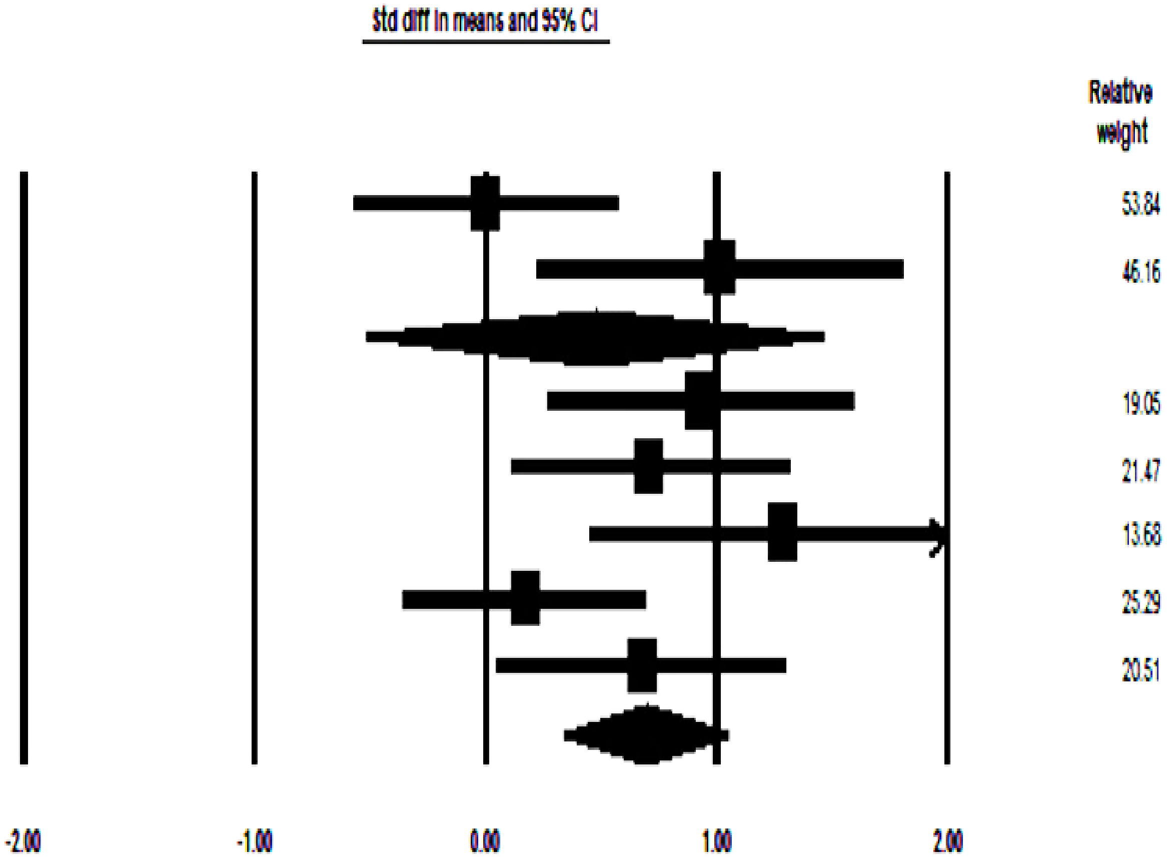

B

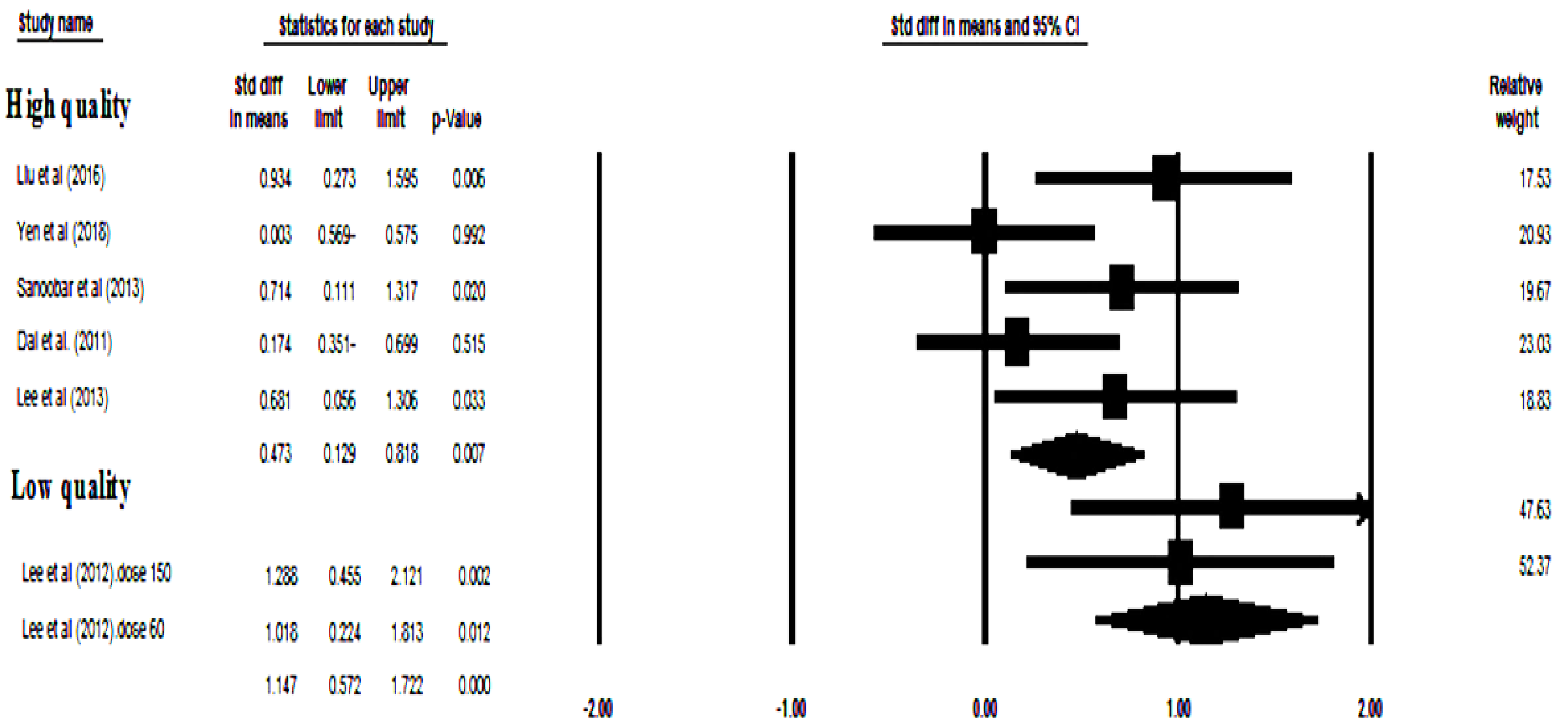

Supplementary figure 8. Subgroup analysis for effect of coenzyme Q10 (CoQ10) on superoxide dismutase (SOD) based on different doses (A. dose ≤ 100 or > 100 mg/d) and studies with different qualities (B. high quality or low quality).
