# Supplementary material for: The effect of coenzyme Q10 supplementation on oxidative stress: A systematic review and meta‐analysis of randomized controlled clinical trials
Source: Food Sci Nutr. 2020 Mar 19;8(4):1766–76. doi: 10.1002/fsn3.1492 (PMC7174219; doi:10.1002/fsn3.1492)
Supplement: Supplementary file 10 — Fig S10 [file FSN3-8-1766-s010.pdf]

A

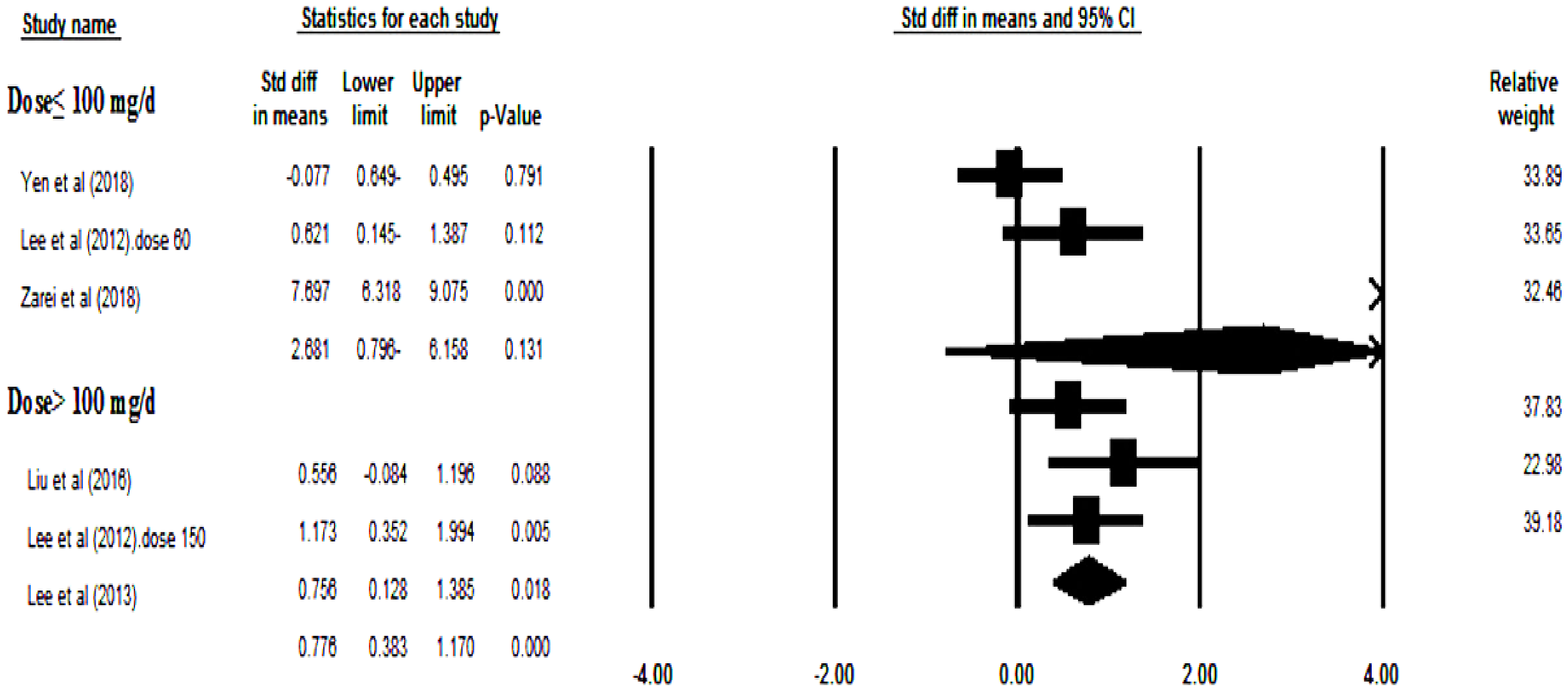

Study name
Statistics for each study
Std diff in means and 95% CI
Dose≤ 100 mg/d
Std diff in means
Lower limit
Upper limit
p-Value
Relative weight
Yen et al (2018) -0.077 0.649- 0.495 0.791 33.89
Lee et al (2012).dose 60 0.621 0.145- 1.387 0.112 33.65
Zarei et al (2018) 7.697 6.318 9.075 0.000 32.46
2.681 0.796- 6.158 0.131
Dose> 100 mg/d
Liu et al (2016) 0.556 -0.084 1.196 0.088 37.83
Lee et al (2012).dose 150 1.173 0.352 1.994 0.005 22.98
Lee et al (2013) 0.756 0.128 1.385 0.018 39.18
0.776 0.383 1.170 0.000
-4.00
-2.00
0.00
2.00
4.00

B

Study name	Std diff in means	Lower limit	Upper limit	p-Value	Relative weight
**High quality**					
Liu et al (2016)	0.556	-0.084	1.196	0.088	25.46
Yen et al (2018)	-0.077	0.649-	0.495	0.791	25.58
Zarei et al (2018)	7.697	6.318	9.075	0.000	23.47
Lee et al (2013)	0.756	0.128	1.385	0.018	25.48
	2.121	0.005	4.237	0.049	
**Low quality**					
Lee et al (2012).dose 60	0.621	0.145-	1.387	0.112	53.47
Lee et al (2012).dose 150	1.173	0.352	1.994	0.005	46.53
	0.878	0.318	1.438	0.002	

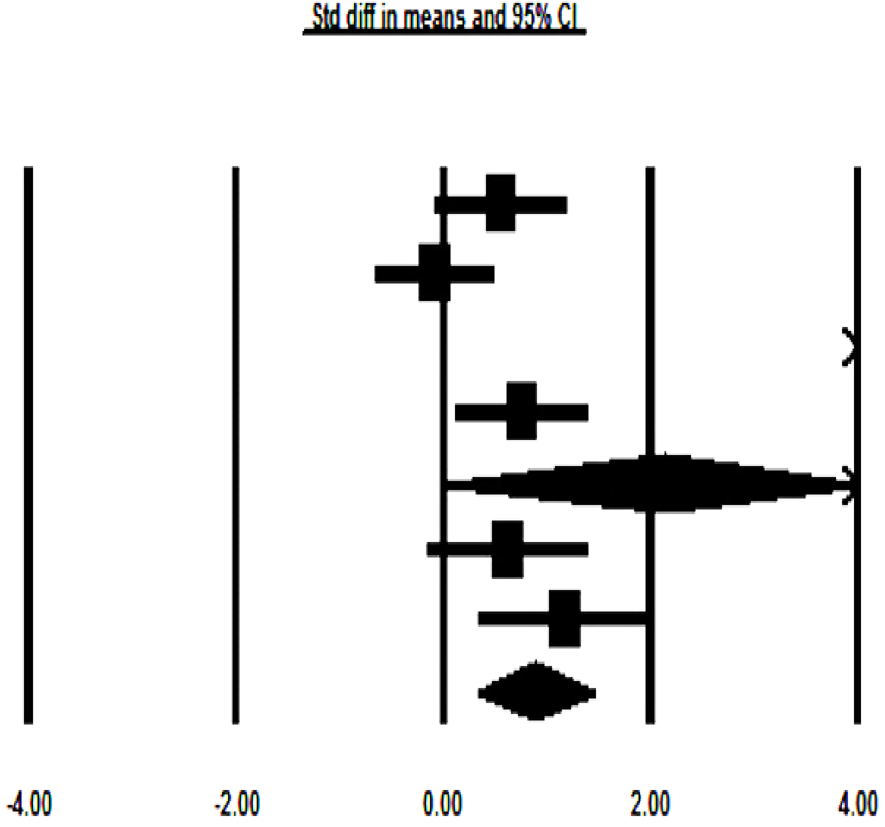

Supplementary figure 10. Subgroup analysis for effect of coenzyme Q10 (CoQ10) on catalse (CAT) level based on the different doses (A. dose ≤ 100 or > 100 mg/d) and studies with different qualities (B. high quality or low quality)
