# Supplementary material for: The effect of coenzyme Q10 supplementation on oxidative stress: A systematic review and meta‐analysis of randomized controlled clinical trials
Source: Food Sci Nutr. 2020 Mar 19;8(4):1766–76. doi: 10.1002/fsn3.1492 (PMC7174219; doi:10.1002/fsn3.1492)
Supplement: Supplementary file 11 — Fig S11 [file FSN3-8-1766-s011.pdf]

A

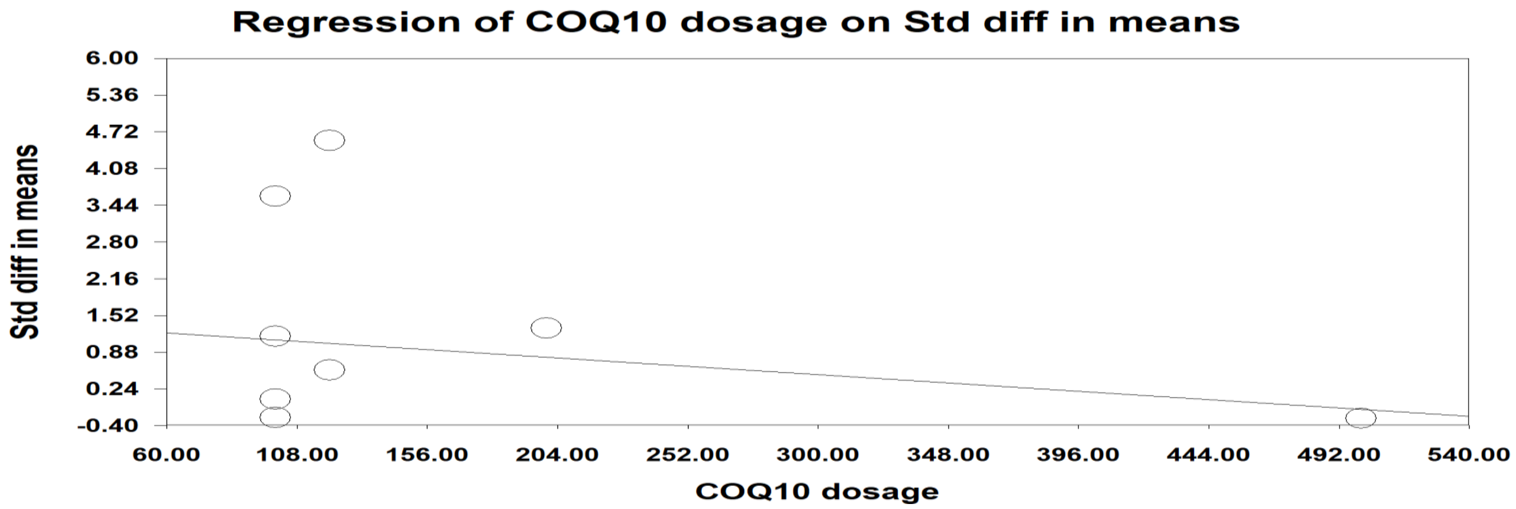

Regression of COQ10 dosage on Std diff in means
Std diff in means
6.00
5.36
4.72
4.08
3.44
2.80
2.16
1.52
0.88
0.24
-0.40
60.00
108.00
156.00
204.00
252.00
300.00
348.00
396.00
444.00
492.00
540.00
COQ10 dosage

B

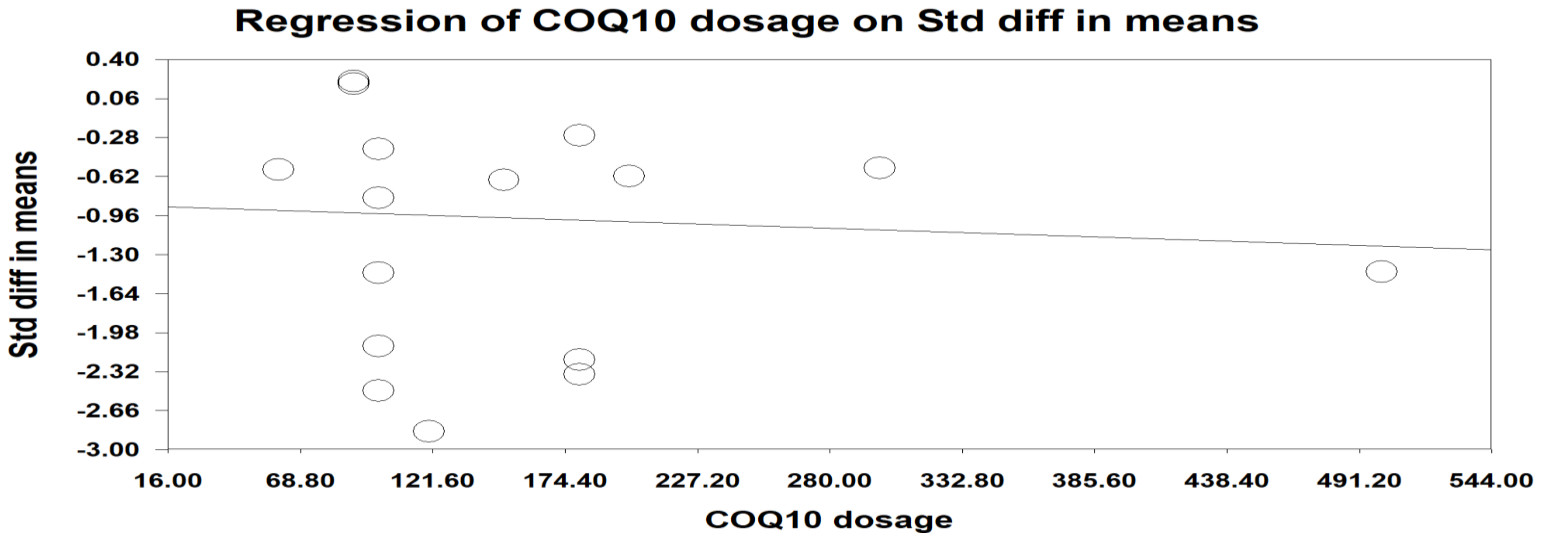

Regression of COQ10 dosage on Std diff in means
Std diff in means
0.40
0.06
-0.28
-0.62
-0.96
-1.30
-1.64
-1.98
-2.32
-2.66
-3.00
16.00
68.80
121.60
174.40
227.20
280.00
332.80
385.60
438.40
491.20
544.00
COQ10 dosage

C

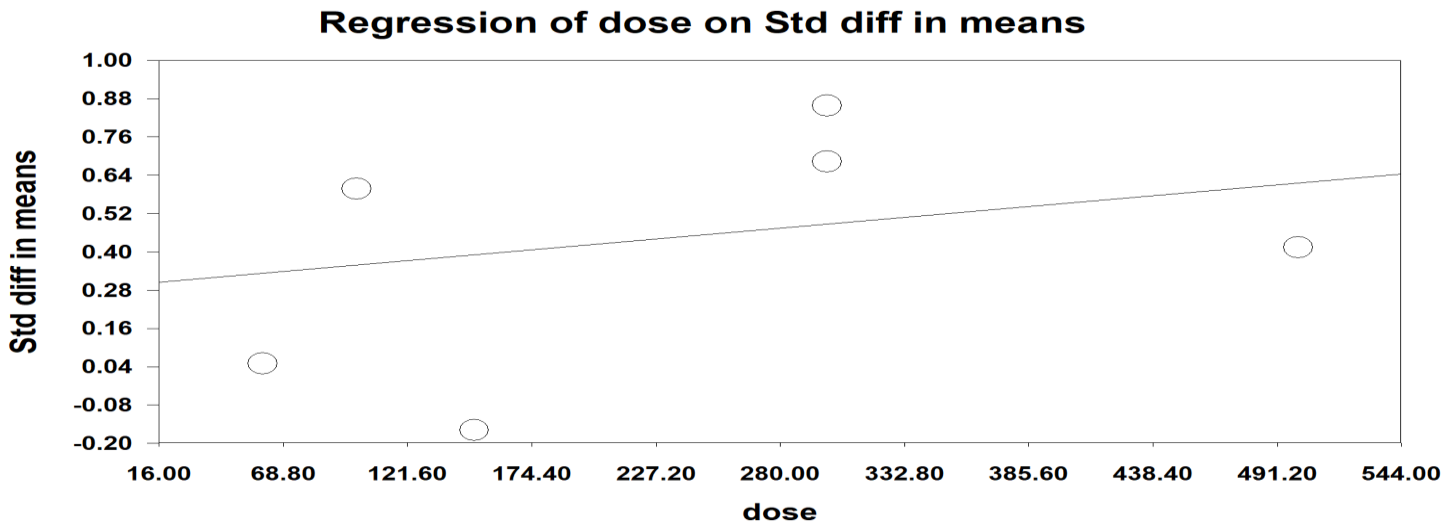
Regression of dose on Std diff in means
Std diff in means
1.00
0.88
0.76
0.64
0.52
0.40
0.28
0.16
0.04
-0.08
-0.20
16.00
68.80
121.60
174.40
227.20
280.00
332.80
385.60
438.40
491.20
544.00
dose

D

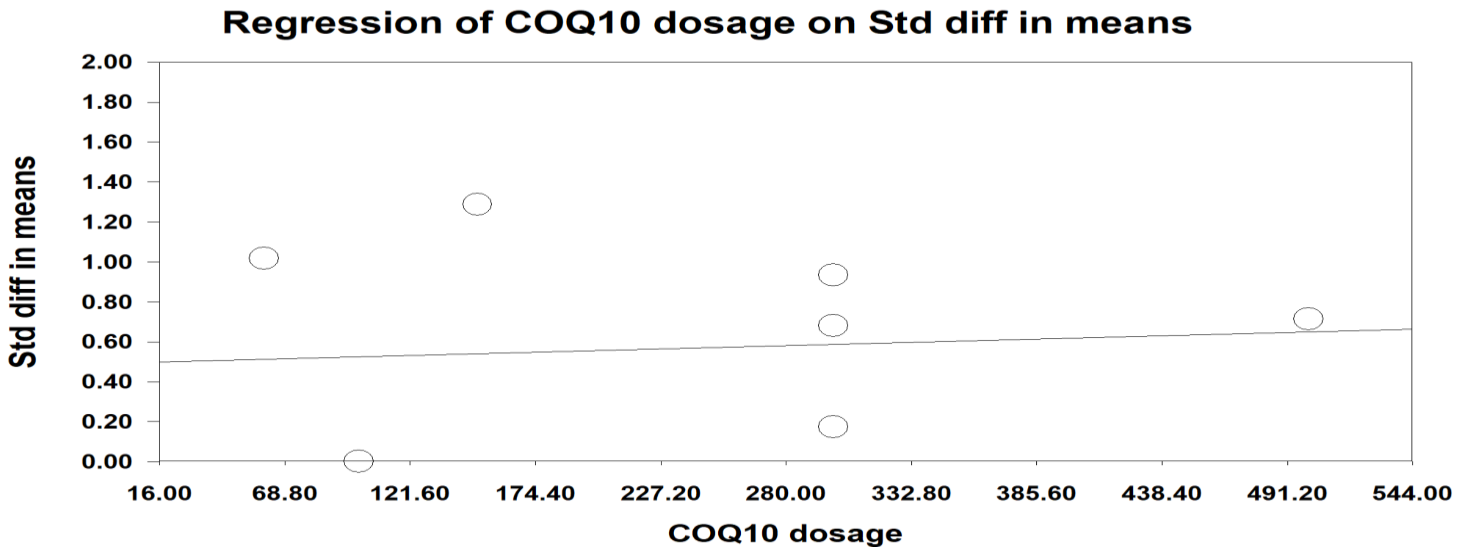
Regression of COQ10 dosage on Std diff in means
Std diff in means
2.00
1.80
1.60
1.40
1.20
1.00
0.80
0.60
0.40
0.20
0.00
16.00
68.80
121.60
174.40
227.20
280.00
332.80
385.60
438.40
491.20
544.00
COQ10 dosage

E

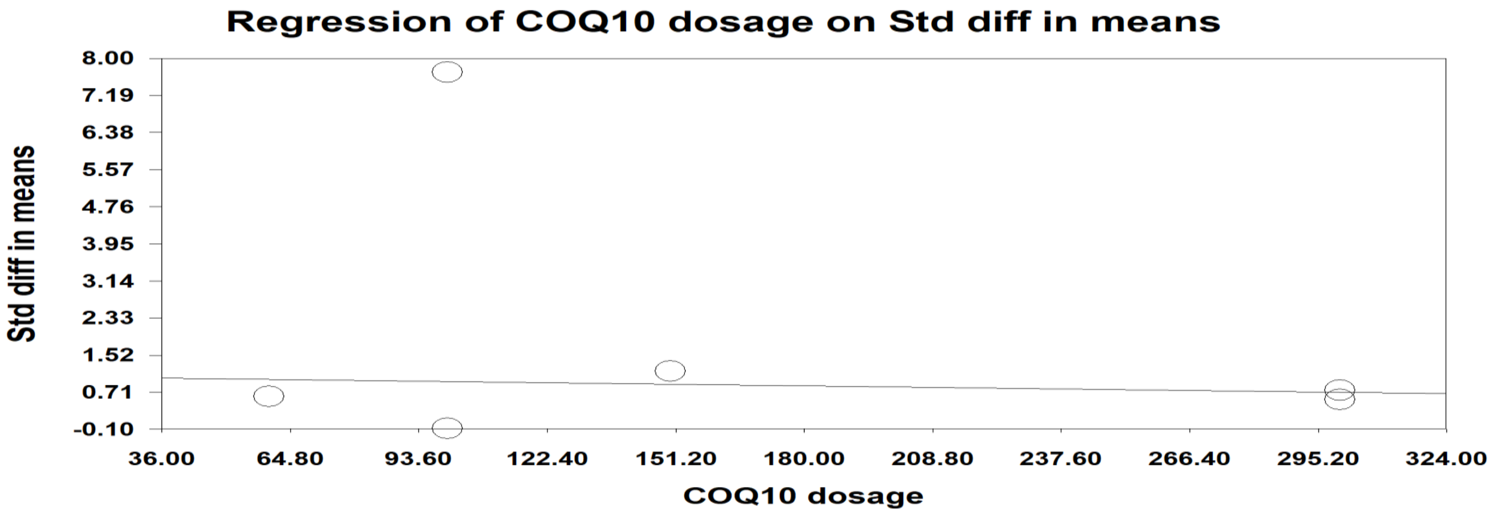

Supplementary Figure 11. Association between standardized mean difference in oxidative stress markers levels values (A: total antioxidant capacity, B: malondialdehyde, C: glutathione peroxidase, D: superoxidase dismutase, E: catalase) after coenzyme Q10 (CoQ10) supplementation with dose of CoQ10.
