# Supplementary material for: The effect of coenzyme Q10 supplementation on oxidative stress: A systematic review and meta‐analysis of randomized controlled clinical trials
Source: Food Sci Nutr. 2020 Mar 19;8(4):1766–76. doi: 10.1002/fsn3.1492 (PMC7174219; doi:10.1002/fsn3.1492)
Supplement: Supplementary file 12 — Fig S12 [file FSN3-8-1766-s012.pdf]

A

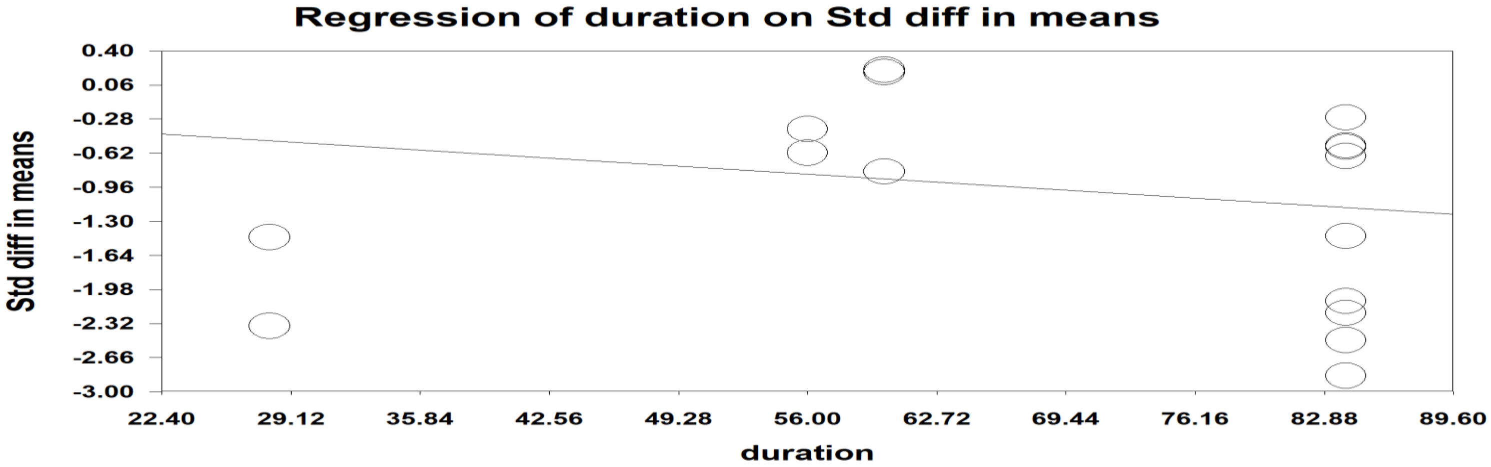

B

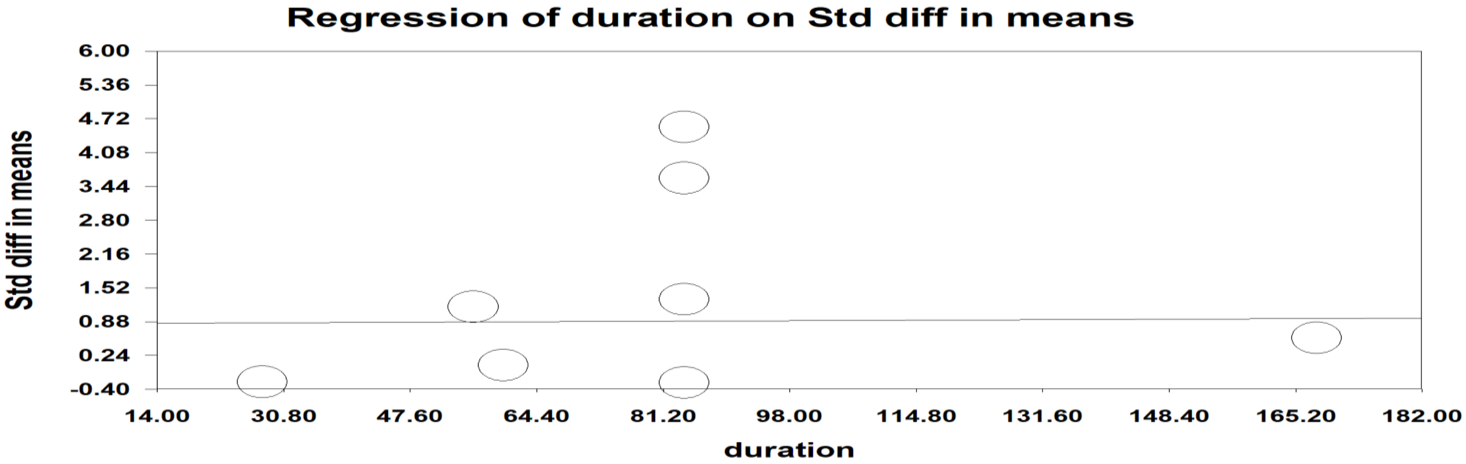

Supplementary Figure 12. Association between standardized mean difference in oxidative stress markers levels values (A: malondialdehyde, B: total antioxidant capacity) after coenzyme Q10 (CoQ10) supplementation with duration of trial.
