# Supplementary material for: The effect of coenzyme Q10 supplementation on oxidative stress: A systematic review and meta‐analysis of randomized controlled clinical trials
Source: Food Sci Nutr. 2020 Mar 19;8(4):1766–76. doi: 10.1002/fsn3.1492 (PMC7174219; doi:10.1002/fsn3.1492)
Supplement: Supplementary file 13 — Fig S13 [file FSN3-8-1766-s013.pdf]

A

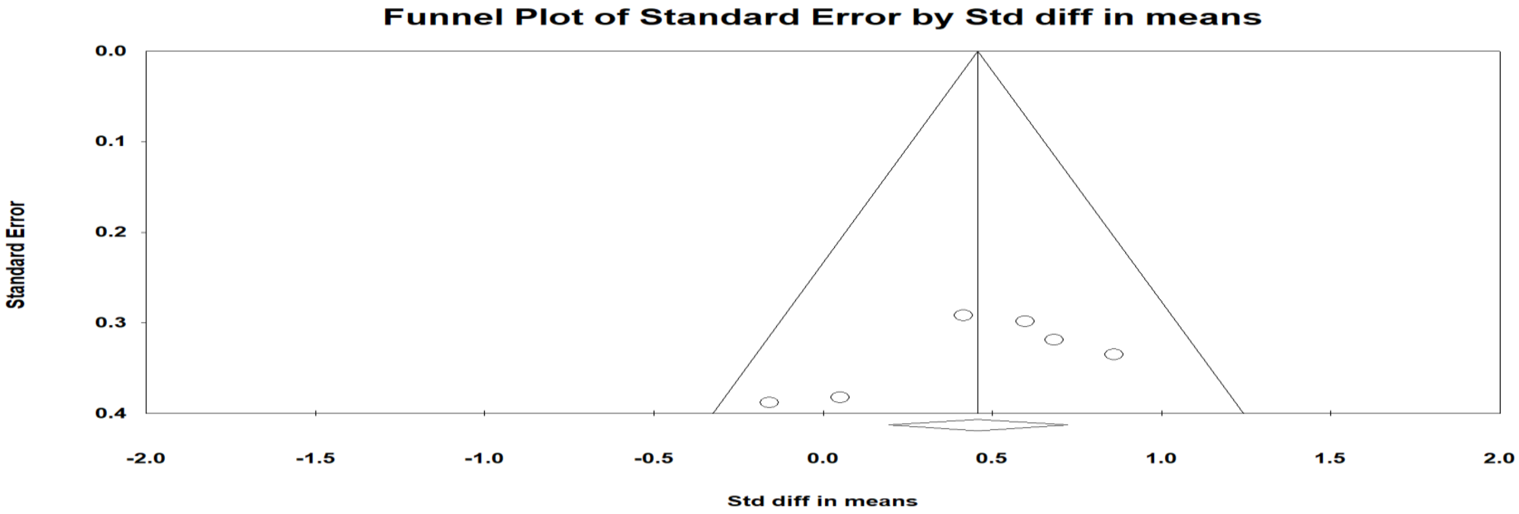

Funnel Plot of Standard Error by Std diff in means
Standard Error
0.0
0.1
0.2
0.3
0.4
-2.0
-1.5
-1.0
-0.5
0.0
0.5
1.0
1.5
2.0
Std diff in means

B

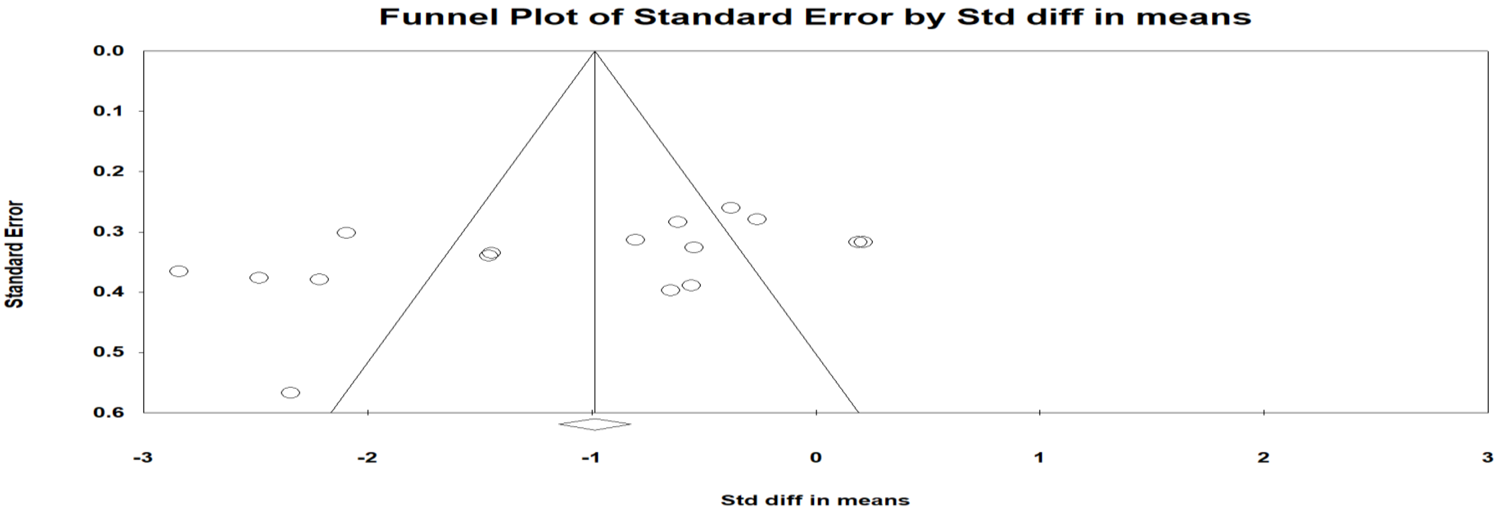

Funnel Plot of Standard Error by Std diff in means
Standard Error
0.0
0.1
0.2
0.3
0.4
0.5
0.6
-3
-2
-1
0
1
2
3
Std diff in means

C

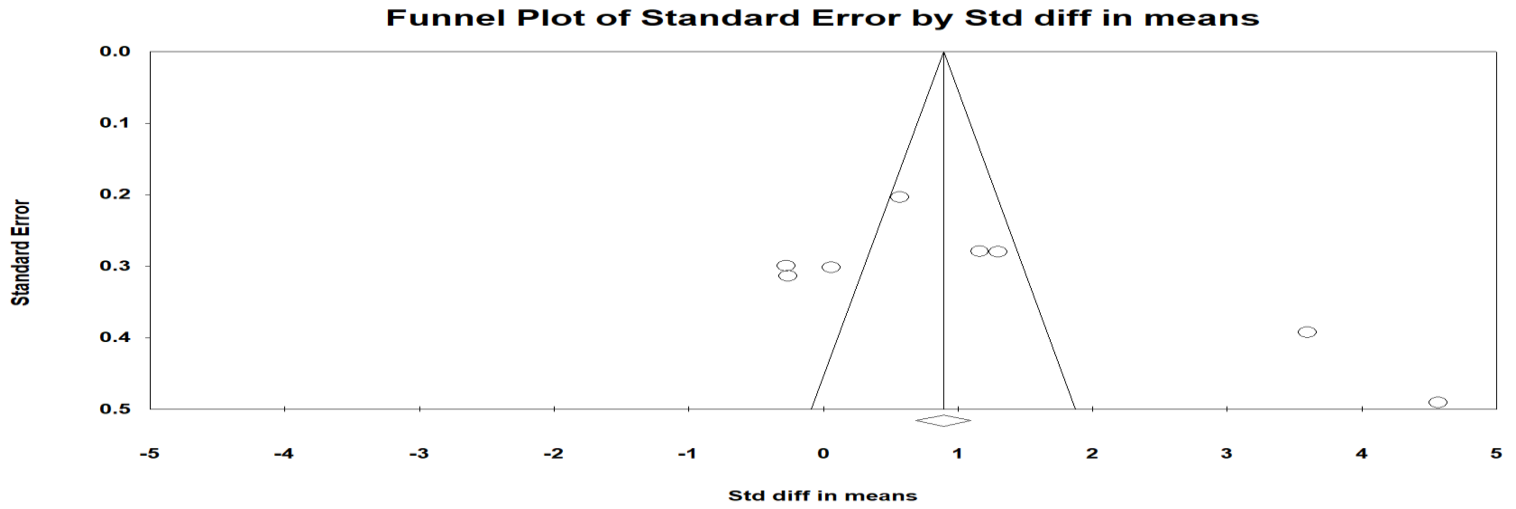

Funnel Plot of Standard Error by Std diff in means
Standard Error
0.0
0.1
0.2
0.3
0.4
0.5
-5
-4
-3
-2
-1
0
1
2
3
4
5
Std diff in means

D

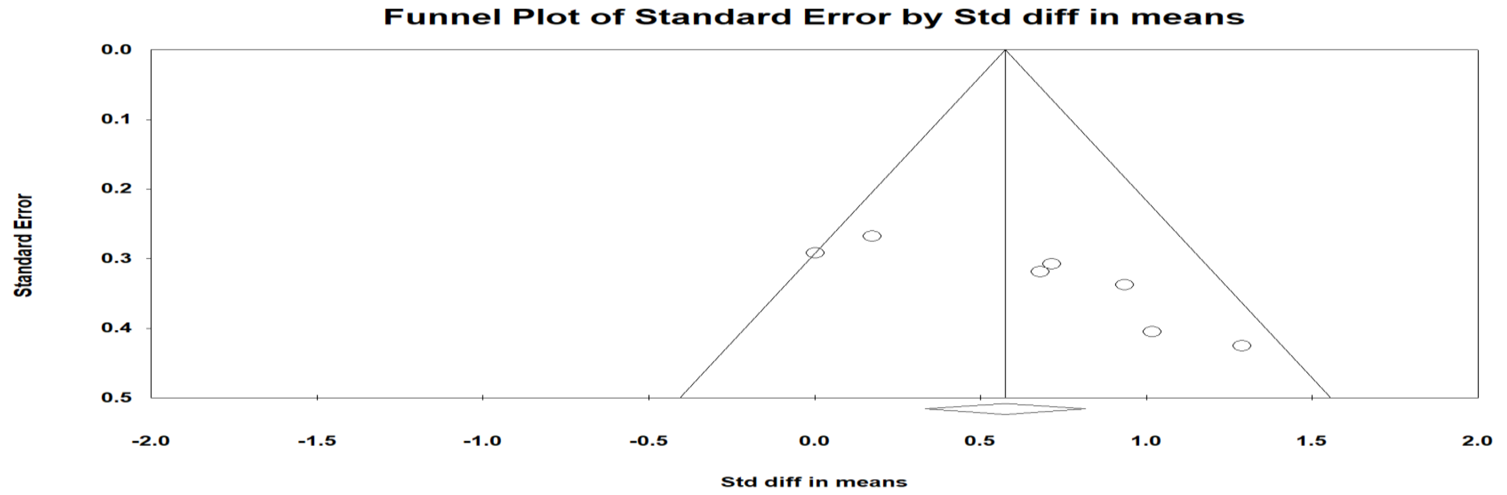

Funnel Plot of Standard Error by Std diff in means
Standard Error
0.0
0.1
0.2
0.3
0.4
0.5
-2.0
-1.5
-1.0
-0.5
0.0
0.5
1.0
1.5
2.0
Std diff in means

E

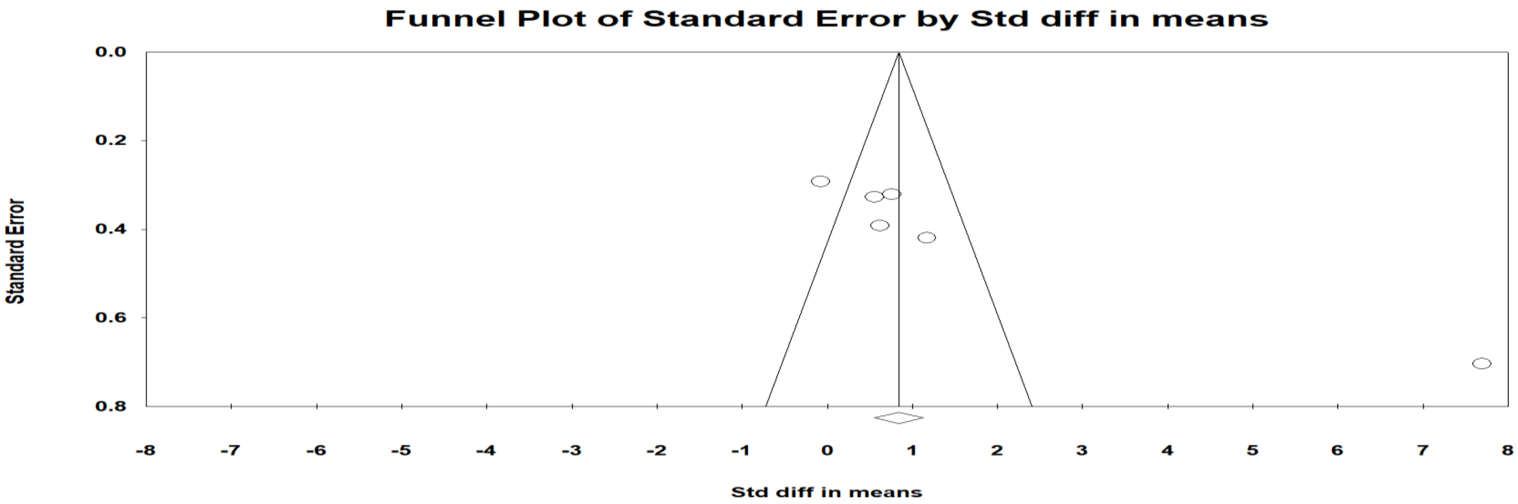

Supplementary Figure 13. Publication bias for oxidative stress markers (A: total antioxidant capacity, B: malondialdehyde, C: glutathione peroxidase, D: superoxidase dismutase, E: catalase) concentrations with coenzyme Q10 (CoQ10)
