# Supplementary material for: The effect of coenzyme Q10 supplementation on oxidative stress: A systematic review and meta‐analysis of randomized controlled clinical trials
Source: Food Sci Nutr. 2020 Mar 19;8(4):1766–76. doi: 10.1002/fsn3.1492 (PMC7174219; doi:10.1002/fsn3.1492)
Supplement: Supplementary file 15 — Table S1 [file FSN3-8-1766-s015.docx]

**Supplementary table 1. Characteristics of studies reporting the effect of coenzyme Q10 (CoQ10) on total antioxidant capacity (TAC) included in the systematic review.**

| **Study** | **Study design** | **Population** | **Intervention** | **Duration** | **CoQ10 group** | | **Placebo group** | | **P-value**  **(Between group)** | **Main**  **outcomes** |
| --- | --- | --- | --- | --- | --- | --- | --- | --- | --- | --- |
|  |  |  |  |  | **^1^B** | **^2^A** | **^1^B** | **^2^A** |  |  |
| Abbasalizad Farhangi  et al (2014) | Randomized double-blind, placebo- controlled trial, parallel | NAFLD patients (Total n=44; Completed study: intervention: 20, placebo: 21) | CoQ10  (100 mg/d) or placebo | 28  days | 1.74 ± 0.038 | 1.60 ± 0.27 | 1.69 ± 0.32 | 1.62 ± 0.052 | **Between groups:**  0.412 | AST, ALT, FSG, IR, vaspin, chemerin, PTX3, TAC, MDA |
| Abdollahzad  et al (2015) | Randomized double-blind, placebo- controlled trial, parallel | RA  patients (Total n=54; Completed study: intervention: 22, placebo: 22) | CoQ10  (100 mg/d) or placebo | 60  days | 0.77 ± 0.55 | 0.96 ± 0.58 | 0.81 ± 0.47 | 0.96 ± 0.47 | **Before intervention:**  0.790  **After intervention:**  0.744 | MDA, TAC,  IL-6, TNF-α |
| Sanoobar  et al (2013) | Randomized double-blind, placebo- controlled trial, parallel | MS  patients  (Total n=48; Completed study: intervention: 22, placebo: 23) | CoQ10  (500 mg/d) or placebo | 84  days | 2204*.*6 ± 352 | 2086*.*2 ± 296 | 2334*.*8 ± 406 | 2298*.*4 ± 379 | **Between groups:**  0*.*294 | MDA,  TAC, SOD, GPx |
| Zhang  et al (2017) | Randomized double-blind, placebo- controlled trial, parallel | Patients with dyslipidemia (Total n=101; Completed study: intervention: 51, placebo: 50) | CoQ10  (120 mg/d) or placebo | 168  days | 0.87 ± 0.19 | 0.99 ± 0.24 | 0.94 ± 0.24 | 0.95 ± 0.25 | **Between groups:**  0.12 | TG, TC, LDL, HDL, SBP, DBP, FBS, insulin, HOMA-IR, hs-CRP, TAC, Non-LDL (non- HDL), ApoA-I, ApoB, ALT, AST, GGT, urea, Cr,  uric acid |
| Raygan  et al (2016) | Randomized double-blind, placebo controlled trial, parallel | T2DM  patients with CHD  (Total n=60; Completed study: intervention: 30, placebo: 30) | CoQ10  (100mg/d) or placebo | 56  days | 834.0 ± 156.4 | 860.1 ± 190.8 | 1156.5 ± 333.9 | 994.3 ± 196.3 | **Between groups:**  0.008 | FPG, Insulin, HOMA-IR, HOMA-B, QUICKI, TG, VLDL, TC, LDL, HDL, hs-CRP, NO, TAC, GSH, MDA |
| Akbari Fakhraba  et al (2014) | Randomized double-blind, placebo-  controlled, parallel | T2DM  patients with neuropathy (Total  n=74; Completed study: intervention: 32, placebo: 30) | CoQ10  (200 mg/d) or placebo | 84  days | 0.007 ± 0.001 | 0.009 ± 0.002 | 0.008 ± 0.002 | 0.008 ± 0.001 | **Between groups:**  0.04 | FBS, lipid profile,  insulin, HbA1C, hs-CRP,  TAC |
| Zarei  et al (2018) | Randomized double-blind, placebo- controlled trial, parallel | T2DM  patients (Total  n=68; Completed study: intervention: 34, placebo: 34) | CoQ10  (100 mg/d) or placebo | 84  days | 0.300 ± 0.012 | 0.340 ± 0.007 | 0.310 ± 0.010 | 0.320 ±0.006 | **Between groups:**  0.003 | HbA1C, FBS, α-amylase, ADA, CAT, TAC, insulin, QUICKI |
| Fallah  et al (2019) | Randomizeddouble-blind, placebo-  controlled, parallel | T2DM  Hemodialysis patients (Total  n=60; Completed study: intervention: 30, placebo: 30) | CoQ10  (120 mg/d) or placebo | 84  days | 650.470±87.829 | 680.765 ±108.302 | 846.079 ±144.443 | 743.923 ±149.011 | **Between groups:**  <0.001 | hs-CRP, NO, TAC, MDA |

^1^B: Before intervention; ^2^A: After intervention. CoQ10: Coenzyme Q10; NAFLD: Non Fatty Liver Disease; PTX3: Pentraxin 3; RA: Rheumatoid Arthritis; MS: Multiple Sclerosis; T2DM: Type 2 Diabetes Mellitus; AST: Aspartate Aminotransferase; ALT: Alanine Aminotransferase; FSG: Fasting Serum Glucose; IR: Insulin Resistance; TAC: Total Antioxidant Capacity; MDA: Malondialdehyde; IL-6: Interleukin 6; TNF-α: Tumor Necrosis Alpha, SOD: Superoxide Dismutase; GPx: Glutathione Peroxidase; TC: Total Cholesterol; LDL: Low Density Lipoprotein; HDL: High Density Lipoprotein; TG: Triglyceride; SBP: Systolic Blood Pressure; DBP: Diastolic Blood Pressure; FBS: Fasting Blood Sugar; HOMA-IR: Homeostasis Model Assessment-Insulin Resistance; hs-CRP: High Sensitivity C-reactive Protein; ApoA-I: [Apolipoprotein A1; Apo-B:](https://www.google.com/url?sa=t&rct=j&q=&esrc=s&source=web&cd=1&cad=rja&uact=8&ved=2ahUKEwjsyJqHkd_gAhXSKlAKHXH2DtYQFjAAegQICRAB&url=https%3A%2F%2Fen.wikipedia.org%2Fwiki%2FApolipoprotein_A1&usg=AOvVaw0-jQju5nOcIQoWw9jh84TB) [Apolipoprotein B; FPG: Fasting Plasma Glucose; HOMA-B: Homeostasis Model Assessment, β-cell function; HbA1C:](https://www.google.com/url?sa=t&rct=j&q=&esrc=s&source=web&cd=1&cad=rja&uact=8&ved=2ahUKEwiE4ra7kd_gAhVDZlAKHem6CrkQFjAAegQICRAB&url=https%3A%2F%2Fen.wikipedia.org%2Fwiki%2FApolipoprotein_B&usg=AOvVaw30liehWrD-pNz6lHuI41wW) [Hemoglobin A1c; ADA: Adenosine deaminase; CAT: Catalase; QUICKI:](https://www.google.com/url?sa=t&rct=j&q=&esrc=s&source=web&cd=1&cad=rja&uact=8&ved=2ahUKEwiRhprmkt_gAhVD16QKHUxlAuwQFjAAegQIChAB&url=https%3A%2F%2Fwww.webmd.com%2Fdiabetes%2Fguide%2Fglycated-hemoglobin-test-hba1c&usg=AOvVaw1b3BeTdIzX-FVOlrwKTuAz) [Quantitative Insulin Sensitivity Check Index; NO: Nitric Oxide; VLDL: Very Low Density Lipoprotein; GSH:](https://www.google.com/url?sa=t&rct=j&q=&esrc=s&source=web&cd=2&cad=rja&uact=8&ved=2ahUKEwi8-pewk9_gAhWF16QKHcdZBtcQFjABegQICRAB&url=https%3A%2F%2Facademic.oup.com%2Fjcem%2Farticle%2F85%2F7%2F2402%2F2851441&usg=AOvVaw1Ppg5thtUl1Lu-7xFLEhmA) [Glutathione; GGT:](https://www.google.com/url?sa=t&rct=j&q=&esrc=s&source=web&cd=1&cad=rja&uact=8&ved=2ahUKEwj2n6j3k9_gAhUC3aQKHZZ8CwcQFjAAegQICxAB&url=https%3A%2F%2Fen.wikipedia.org%2Fwiki%2FGlutathione&usg=AOvVaw25nLLayE-z9n4Ti_v3ZJlv) [Gamma-Glutamyl Transferase; Cr: creatinine; CHD: coronary heart disease. All values have been presented as mean±SD.](https://www.google.com/url?sa=t&rct=j&q=&esrc=s&source=web&cd=1&cad=rja&uact=8&ved=2ahUKEwiJu4OjlN_gAhUJqaQKHYMaBg4QFjAAegQIChAB&url=https%3A%2F%2Flabtestsonline.org%2Ftests%2Fgamma-glutamyl-transferase-ggt&usg=AOvVaw1mw0N0Bm1Mj0cSOfggcEcD)
