# Supplementary material for: The effect of coenzyme Q10 supplementation on oxidative stress: A systematic review and meta‐analysis of randomized controlled clinical trials
Source: Food Sci Nutr. 2020 Mar 19;8(4):1766–76. doi: 10.1002/fsn3.1492 (PMC7174219; doi:10.1002/fsn3.1492)
Supplement: Supplementary file 16 — Table S2 [file FSN3-8-1766-s016.docx]

**Supplementary table 2. Characteristics of studies reporting the effect of coenzyme Q10 (CoQ10) on Malondialdehyde (MDA) included in the systematic review.**

| **Study** | **Study**  **design** | **Population** | **Intervention** | **Duration** | **CoQ10 group** | | **Placebo group** | | **P-value**  **(Between group)** | **Main**  **outcomes** |
| --- | --- | --- | --- | --- | --- | --- | --- | --- | --- | --- |
|  |  |  |  |  | **^1^B** | **^2^A** | **^1^B** | **^2^A** |  |  |
| Abbasalizad  et al (2014) | Randomized double-blind, placebo- controlled trial, parallel | NAFLD patients  (Total n=44;  Completed study: intervention: 22; placebo: 22) | CoQ10  (100 mg/d) or placebo | 28 days | 0. 005±0. 002 | 0. 004±0. 002 | 0. 005±0. 002 | 0. 005±0. 001 | **Before intervention:**  0.367  **After intervention:**  0.107 | AST, ALT, FSG, IR, vaspin, chemerin, PTX3, TAC, MDA |
| Abdollahzad  et al (2015) | Randomized double-blind, placebo- controlled trial, parallel | RA patients  (Total n=54; Completed study: intervention: 22; placebo: 22) | CoQ10  (100 mg/d) or placebo | 60 days | 0.004±0.002 | 0.002±0.001 | 0.003±0.002 | 0.003±0.002 | **Before intervention:**  0.274  **After intervention:**  0.006 | MDA, TAC, IL-6, TNF-α |
| Lee  et al (2012) | Randomized double-blind, placebo- controlled trial, parallel | CAD patients  (Total n=32; Completed study: intervention: 14; placebo: 17) | CoQ10  (150 mg/d) or placebo | 84 days | 48.95±17.96 | 42±14 | 47±17 | 50±21 | **Between groups:**  0.03 | BUN, Cr, TC, TG,  LDL, HDL, hs-CRP, P COQ10, MDA, SOD, GPx, CAT |
| Lee  et al (2012) | Randomized double-blind, placebo- controlled trial, parallel | CAD patients  (Total n=33; Completed study: intervention: 10; placebo: 17) | CoQ10  (60 mg/d) or placebo | 84 days | 54±20 | 48±10 | 47±17 | 50±21 | **Between groups:**  Not significant | BUN, Cr, TC, TG,  LDL, HDL, hs-CRP, IL-6, COQ10, MDA, SOD, homocysteine |
| Sanoobar  et al (2013) | Randomized double-blind, placebo- controlled trial, parallel | MS patients  (Total n=48; Completed study: intervention: 22; placebo: 23) | CoQ10  (500 mg/d) or placebo | 84 days | 1*.*97±0*.*5 | 1*.*630±0*.*3 | 1*.*91±0*.*3 | 2*.*08 ±0*.*4 | **Between groups:**  0.03 | MDA,  TAC,SOD, GPx |
| Liu  et al (2016) | Randomized double-blind, placebo- controlled trial, parallel | HCC patients  (Total n=41; Completed study: intervention: 20; placebo: 19) | CoQ10  (300 mg/d) or placebo | 84 days | 0.001±0.001 | 0.001±0.00031 | 0.001±0.00034 | 0.001±0.0003 | **Between groups:**  P < 0.01 | COQ10, Vit E, hs-CRP, IL-6, BUN, Cr, GOT, GPT, TC, TG, LDL, HDL, TC / HDL, TNF-α, MDA, SOD, CAT, GPx |
| Gholami  et al (2018) | Randomized double-blind, placebo- controlled trial, parallel | T2DM patients  (Total n=68; Completed study: intervention: 34; placebo: 34) | CoQ10  (100 mg/d) or placebo | 84 days | 0.011± 0.001 | 0.001± 0.00034 | 0.012±0.001 | 0.011±0.00005 | **Before intervention:**  0.571  **After intervention:**  0.006 | FBG, HbA1C, Insulin, TC, TG, HDL, LDL, CoQ10, CoQ10/TC, Adiponectin, Leptin, MDA, 8-Isoprostan, |
| Gholnari  et al (2017) | Randomized double-blind, placebo- controlled trial, parallel | DN patients  (Total n=50; Completed study: intervention: 25; placebo: 25) | CoQ10  (100 mg/d) or placebo | 84 days | 0.004 ±0.002 | 0.003 ±0.001 | 0.003±0.001 | 0.004±0.001 | **Between groups:**  <0.001 | FPG, Insulin, HOMA-IR  HOMA-B, QUICKI, HbA1c, TG, VLDL, TC, LDL, HDL, MMP-2, MDA, AGEs, CG, Cr, BUN |
| Raygan  et al (2016) | Randomized double-blind, placebo- controlled trial, parallel | T2DM patients with CHD  (Total n= 60; Completed study: intervention: 30; placebo: 30) | CoQ10  (100 mg/d) or placebo | 56 days | 0.005±0.001 | 0.005±0.001 | 0.005±0.002 | 0.005±0.002 | **Between groups:**  0.008 | FPG, Insulin, HOMA-IR, HOMA-B, QUICKI, TG, VLDL, TC, LDL, HDL, hs-CRP, NO, TAC, GSH, MDA |
| Singh  et al (2000) | Randomized double-blind, placebo- controlled trial | CRF patients  (Total n=21; Completed study: intervention: 11; placebo: 10) | CoQ10  (180 mg/d) or placebo | 28 days | 0.000004±0.000001 | 0.000002±0.0000008 | 0.000004±0.000001 | 0.000004±0.000001 | **Between groups:**  p<0.05 | Vitamins A&C&E, Beta-carotene, Diene conjugate, VitE/cholesterol |
| Kaikkonen et al (1996) | Randomized double-blind, placebo- controlled trial, parallel | Healthy men  (Total n=40; Completed study: intervention: 20; placebo: 20) | Granule CoQ10  (90 mg/d) or placebo | 60 days | 0.0003±0.0001 | Not reported | 0.0004±0.0001 | Not reported | **Between groups:**  0.83 | Plasma Q10, VLDL+LDL fraction,  Lag time, Vmax  LDL, MDA,  ascorbate, a-tocopherol |
| Kaikkonen et al (1996) | Randomized double-blind, placebo- controlled trial, parallel | Healthy men  (Total n=40; Completed study: intervention: 20; placebo: 20) | oil-based CoQ10  (90 mg/d) or placebo | 60 days | 0.0004 ±0.0001 | Not reported | 0.0004±0.0001 | Not reported | **Between groups:**  0.83 | Plasma Q10, VLDL+LDL fraction,  Lag time, Vmax  LDL, MDA,  ascorbate, a-tocopherol |
| Moazen  et al (2015) | Randomized double-blind, placebo- controlled trial, parallel | T2DM patients  (Total n=52; Completed study: intervention: 26; placebo: 26) | CoQ10  (200 mg/d) or placebo | 56 days | 0.013±0.006 | 0.01±0.003 | 0.011±0.003 | 0.012±0.005 | **Between groups:**  0.19 | FBS, HbA1c, MDA,  Adiponectin |
| Singh  et al (2003) | Randomized double-blind, placebo- controlled trial, parallel | ESRF Patients with HD  (Total n=97; Completed study: intervention: 21; placebo: 24) | CoQ10  (180 mg/d) or placebo | 84 days | 0.003±0.0004 | 0.002±0.0004 | 0.003±0.0004 | 0.003±0.001 | **Between groups:**  p<0.001 | BUN, glucose, Cr,  ẞcarotene, TBARS, diene conjugates, MDA |
| Singh  et al (2003) | Randomized double-blind, placebo- controlled trial, parallel | ESRF Patients without HD  (Total n=97; Completed study: intervention: 27; placebo: 25) | CoQ10  (180 mg/d) or placebo | 84 days | 0.003±0.001 | 0.002±0.001 | 0.003±0.004 | 0.003±0.005 | **Between groups:**  p<0.001 | BUN, glucose, Cr,  ẞcarotene, TBARS, diene conjugates, MDA |
| Fallah  et al (2019) | Randomized double-blind, placebo- controlled trial, | T2D patients with HD  (Total n=60; Completed study: intervention: 30; placebo: 30) | CoQ10  (120 mg/d) or placebo | 84 days | 0.015±0.006 | 0.003± 0.0002 | 0.003±0.001 | 0.004±0.003 | **Between groups:**  0.09 | TAC, GSH, MDA, hs-CRP,NO |

.

^1^B: Before intervention; ^2^A: After intervention. CoQ10: Coenzyme Q10; HCC: hepatocellular carcinoma; NAFLD: Non Fatty Liver Disease; AST: Aspartate Aminotransferase; ALT: Alanine Aminotransferase; FSG: Fasting Serum Glucose; ; IR: Insulin Resistance; PTX3: Pentraxin 3; TAC: Total Antioxidant Capacity; MDA: Malondialdehyde; RA: Rheumatoid Arthritis; IL-6: Interleukin 6; TNF-α: Tumor Necrosis Alpha, CAD: coronary artery disease; BUN: Blood urea nitrogen; Cr: creatinine; TC: Total Cholesterol; TG: Triglyceride; LDL: Low Density Lipoprotein; HDL: High Density Lipoprotein; hs-CRP: High Sensitivity C-reactive Protein; SOD: Superoxide Dismutase; GPx: Glutathione Peroxidase; CAT: Catalase; MS: Multiple Sclerosis; HCC: hepatocellular carcinoma; GOT: glutamic oxaloacetic transaminase; GPT: glutamic pyruvic transaminase; T2DM: Type 2 Diabetes Mellitus; FSG: Fasting Serum Glucose; HbA1C: Hemoglobin A1c; DN : Diabetic nephropathy; FPG: Fasting Plasma Glucose; HOMA-IR, homeostasis model of assessment-estimated insulin resistance; HOMA-B, homeostasis model of assessment-estimated B cell function; QUICKI, quantitative insulin sensitivity check index.; MMP-2, matrix metalloproteinase-2; AGEs: advanced glycation end products; CG, Cockcroft-Gault formula (to estimate of creatinine clearance); NO: Nitric Oxide; GSH: Glutathione; Vmax: Maximal velocity; FBS: Fasting Blood Sugar; ESRF: End stage of renal failure; HD: hemodialysis; TBARS: thiobarbituric acid reactive substances. All values have been presented as mean±SD.
