# Supplementary material for: The effect of coenzyme Q10 supplementation on oxidative stress: A systematic review and meta‐analysis of randomized controlled clinical trials
Source: Food Sci Nutr. 2020 Mar 19;8(4):1766–76. doi: 10.1002/fsn3.1492 (PMC7174219; doi:10.1002/fsn3.1492)
Supplement: Supplementary file 17 — Table S3 [file FSN3-8-1766-s017.docx]

**Supplementary table 3. Characteristics of studies reporting the effect of coenzyme Q10 (CoQ10) on Glutathione Peroxidase (GPx) included in the systematic review.**

| **Study** | **Study design** | **Population** | **Intervention** | **Duration** | **CoQ10 group** | | **Placebo group** | | **P-value**  **(Between groups)** | **Main**  **outcomes** |
| --- | --- | --- | --- | --- | --- | --- | --- | --- | --- | --- |
|  |  |  |  |  | **^1^B** | **^2^A** | **^1^B** | **^2^A** |  |  |
|  |  |  |  |  |  |  |  |  |  |  |
| Sanoobar  et al (2013) | Randomized double-blind, placebo- controlled trial, parallel | MS patients  ( Total n=48; Completed study: intervention: 24, placebo: 24) | CoQ10  (500 mg/d) or placebo | 84 days | 264.7±62 | 260±44 | 272.8± 63 | 244 ±45 | **Between groups:**  0.332 | MDA,TAC, SOD, GPx |
| Lee  et al (2013) | Randomized double-blind, placebo- controlled trial, parallel | CAD patients  ( Total n=51; Completed study: intervention: 23, placebo: 19) | CoQ10  (300 mg/d) or placebo | 84 days | 22±3 | 26±4 | 20±4 | 23±5 | **Between groups:**  0.042 | Cr, TC, TG, LDL,  HDL, hs-CRP, TNF-α, IL-6, adiponectin, COQ10, Vit E, SOD, GPx, |
| Yen  et al (2018) | Randomized double-blind, placebo- controlled trial, parallel | T2DM patients  (Total n=50; Completed study: intervention: 24, placebo: 23) | liquid ubiquinol  (100 mg/d) or placebo | 84 days | 25.78±7.45 | 29.95±7.21 | 28.05±5.45 | 28.05±5.45 | **Between groups:**  0.03 | COQ10, FSG, HOMA-IR, lipid profiles, oxidative stress and anti-oxidative enzyme activities |
| Liu  et al (2016) | Randomized double-blind, placebo- controlled trial, parallel | HCC patients  (Total n= 41; Completed study: intervention: 20, placebo: 19) | CoQ10  (300 mg/d) or placebo | 84 days | 15.94±4.77 | 19.4±8.34 | 15.22±5.76 | 12.73±5.46 | **Between groups:**  p < 0.01 | COQ10, Vit E, hs-CRP, IL-6, BUN, Cr, GOT, GPT, TC, TG, LDL, HDL, TC / HDL, TNF-α, MDA, SOD, CAT, GPx |
| Lee  et al (2012) | Randomized double-blind, placebo- controlled trial, parallel | CAD patients  (Total n =32; Completed study: intervention: 15, placebo: 12) | CoQ10  (150 mg/d) or placebo | 84 days | 2.7±1 | 2.3±1.4 | 2.8±9 | 3.3±1.7 | **Between groups:**  Not significant | BUN, Cr, TC, TG, LDL, HDL, hs-CRP, IL-6, COQ10, MDA, SOD, homocysteine |
| Lee  et al (2012) | Randomized double-blind, placebo- controlled trial, parallel | CAD patients  (Total n =32; Completed study: intervention: 16, placebo: 12) | CoQ10  (60 mg/d) or placebo | 84 days | 2.6±8 | 3.5±1.7 | 2.8±9 | 3.3±1.7 | **Between groups:**  Not significant | BUN, Cr, TC, TG, LDL, HDL, hs-CRP, IL-6, COQ10, MDA, SOD, homocysteine |

^1^B: Before intervention; ^2^A: After intervention. CoQ10: Coenzyme Q10; MS: Multiple Sclerosis; MDA: Malondialdehyde; TAC: Total Antioxidant Capacity; SOD: Superoxide Dismutase; GPx: Glutathione Peroxidase; CAD: coronary artery disease; Cr: creatinine; TC: Total Cholesterol; TG: Triglyceride; LDL: Low Density Lipoprotein; HDL: High Density Lipoprotein; hs-CRP: High Sensitivity C-reactive Protein; TNF-α: Tumor Necrosis Alpha; IL-6: Interleukin 6; T2DM: Type 2 Diabetes Mellitus; FSG: Fasting Serum Glucose; HOMA-IR: Homeostasis Model Assessment-Insulin Resistance; HCC: hepatocellular carcinoma; BUN: blood urea nitrogen; GOT: glutamic oxaloacetic transaminase; GPT: glutamic pyruvic transaminase; [CAT: Catalase. All values have been presented as mean±SD.](https://www.google.com/url?sa=t&rct=j&q=&esrc=s&source=web&cd=1&cad=rja&uact=8&ved=2ahUKEwiRhprmkt_gAhVD16QKHUxlAuwQFjAAegQIChAB&url=https%3A%2F%2Fwww.webmd.com%2Fdiabetes%2Fguide%2Fglycated-hemoglobin-test-hba1c&usg=AOvVaw1b3BeTdIzX-FVOlrwKTuAz)
