# Supplementary material for: The effect of coenzyme Q10 supplementation on oxidative stress: A systematic review and meta‐analysis of randomized controlled clinical trials
Source: Food Sci Nutr. 2020 Mar 19;8(4):1766–76. doi: 10.1002/fsn3.1492 (PMC7174219; doi:10.1002/fsn3.1492)
Supplement: Supplementary file 18 — Table S4 [file FSN3-8-1766-s018.docx]

**Supplementary table 4. Characteristics of studies reporting the effect of coenzyme Q10 (CoQ10) on Superoxide Dismutase (SOD) included in the systematic review.**

| **Study** | **Study design** | **Population** | **Intervention** | **Duration** | **CoQ10 group** | | **Placebo group** | | **P-value**  **(Between group)** | **Main**  **outcomes** |
| --- | --- | --- | --- | --- | --- | --- | --- | --- | --- | --- |
|  |  |  |  |  | **^1^B** | **^2^A** | **^1^B** | **^2^A** |  |  |
| Dai  et al (2011) | Randomized double-blind, placebo- controlled trial, parallel | ischemic LVSD patients (Total n=56; Completed study: intervention: 28, placebo: 28) | CoQ10  (300 mg/d) or placebo | 56 days | 0.0001±0.00004 | Not reported | 0.0001±0.00004 | Not reported | **Before intervention:**  0.75  **After intervention:**  0.38 | FBS, HbA1c, TG, LDL, HDL, TC, hs-CRP, SBP, DBP, Cr, ApoA-I, ApoB, LPa, lactate, pyruvate, LP Ratio, SOD, 8-isoprostane, FMD |
| Lee et  al (2012) | Randomized double-blind, placebo- controlled trial, parallel | CAD patients  (Total n=32; Completed study: intervention: 15, placebo: 12) | CoQ10  (150 mg/d) or placebo | 84 days | 26.34±15.36 | 32.61±16.90 | 31.12±9.34 | 18.29 ±11.21 | **Between groups:**  0.34 | BUN, Cr, TC, TG, LDL, HDL, hs-CRP, IL-6, COQ10, MDA, SOD, homocysteine |
| Lee  Et al (2012) | Randomized double-blind, placebo- controlled trial, parallel | CAD patients  (Total n=32; Completed study: intervention: 16, placebo: 12) | CoQ10  (60 mg/d) or placebo | 84 days | 22.34±12.36 | 22.61±13.9 | 31.12±9.34 | 18.29 ± 11.21 | **Between groups:**  significant | BUN, Cr, TC, TG, LDL, HDL, hs-CRP, IL-6, COQ10, MDA, SOD, homocysteine |
| Sanoobar  et al (2013) | Randomized double-blind, placebo- controlled trial, parallel | MS patients  (Total n=48; Completed study: intervention: 22, placebo: 23) | CoQ10  (500 mg/d) or placebo | 84 days | 0.184±0.039 | 0.211±0.06 | 0.186±0.034 | 0.178±0.043 | **Between groups**:  0.013 | MDA, TAC, SOD, GPx activity |
| Lee  et al (2013) | Randomized double-blind, placebo- controlled trial, parallel | CAD patients  (Total n=51; Completed study: intervention: 23, placebo: 19) | CoQ10  (300 mg/d) or placebo | 84 days | 34±11 | 49±19 | 34±14 | 36 ±16 | **Between groups**:  0.034 | Cr, TC, TG, LDL, HDL, hs-CRP, TNF-α, IL-6, adiponectin, COQ10, Vit E, SOD, GPx, CAT |
| Liu  et al (2016) | Randomized double-blind, placebo- controlled trial, parallel | HCC  patients  (Total n=41; Completed study: intervention: 20, placebo: 19) | CoQ10  (300 mg/d) or placebo | 84 days | 13.93 ±4.35 | 21.26 ±7.86 | 16.94 ±8.78 | 17.12 ±5.79 | **Between groups**:  0/01 | COQ10, Vit E, hs-CRP, IL-6, BUN, Cr, GOT, GPT, TC, TG, LDL, HDL, TC / HDL, TNF-α, MDA, SOD, CAT, GPx |
| Yen  et al (2018) | Randomized double-blind, placebo- controlled trial, parallel | T2DM patients  (Total n=50; Completed study: intervention: 24, placebo: 23) | CoQ10  (100 mg/d) or placebo | 84 days | 27.35±17.45 | 22.85±9.45 | 26.5±612.57 | 20.75±7.34 | **Between groups**:  0.01 | COQ10, glucose homoeostasis parameters, lipid profiles, oxidative stress and anti-oxidative enzyme activities |

^1^B: Before intervention; ^2^A: After intervention. CoQ10: Coenzyme Q10; LVSD: left ventricular ejection fraction; FBS: Fasting Blood Sugar; HbA1C: [Hemoglobin A1c; TG: Triglyceride; LDL: Low Density Lipoprotein; HDL: High Density Lipoprotein; TC: Total Cholesterol; hs-CRP: High Sensitivity C-reactive Protein; SBP: Systolic Blood Pressure; DBP: Diastolic Blood Pressure; Cr: creatinine; ApoA-I:](https://www.google.com/url?sa=t&rct=j&q=&esrc=s&source=web&cd=1&cad=rja&uact=8&ved=2ahUKEwiRhprmkt_gAhVD16QKHUxlAuwQFjAAegQIChAB&url=https%3A%2F%2Fwww.webmd.com%2Fdiabetes%2Fguide%2Fglycated-hemoglobin-test-hba1c&usg=AOvVaw1b3BeTdIzX-FVOlrwKTuAz) [Apolipoprotein A1; Apo-B:](https://www.google.com/url?sa=t&rct=j&q=&esrc=s&source=web&cd=1&cad=rja&uact=8&ved=2ahUKEwjsyJqHkd_gAhXSKlAKHXH2DtYQFjAAegQICRAB&url=https%3A%2F%2Fen.wikipedia.org%2Fwiki%2FApolipoprotein_A1&usg=AOvVaw0-jQju5nOcIQoWw9jh84TB) [Apolipoprotein B; LPa: lipoprotein a; SOD: Superoxide Dismutase; FMD: flow-mediated dilatation; CAD: coronary artery disease; BUN: blood urea nitrogen; Cr: creatinine; ; IL-6: Interleukin 6; MDA: Malondialdehyde; TNF-α: Tumor Necrosis Alpha MS: Multiple Sclerosis; TAC: Total Antioxidant Capacity; GPx: Glutathione Peroxidase; TNF-α: Tumor Necrosis Alpha; CAT: Catalase; HCC: hepatocellular carcinoma; GOT: glutamic oxaloacetic transaminase; GPT:](https://www.google.com/url?sa=t&rct=j&q=&esrc=s&source=web&cd=1&cad=rja&uact=8&ved=2ahUKEwiE4ra7kd_gAhVDZlAKHem6CrkQFjAAegQICRAB&url=https%3A%2F%2Fen.wikipedia.org%2Fwiki%2FApolipoprotein_B&usg=AOvVaw30liehWrD-pNz6lHuI41wW)[glutamic pyruvic transaminase; T2DM: Type 2 Diabetes Mellitus. All values have been presenred as mean±SD.](https://www.google.com/url?sa=t&rct=j&q=&esrc=s&source=web&cd=1&cad=rja&uact=8&ved=2ahUKEwiE4ra7kd_gAhVDZlAKHem6CrkQFjAAegQICRAB&url=https%3A%2F%2Fen.wikipedia.org%2Fwiki%2FApolipoprotein_B&usg=AOvVaw30liehWrD-pNz6lHuI41wW)
