# Supplementary material for: The effect of coenzyme Q10 supplementation on oxidative stress: A systematic review and meta‐analysis of randomized controlled clinical trials
Source: Food Sci Nutr. 2020 Mar 19;8(4):1766–76. doi: 10.1002/fsn3.1492 (PMC7174219; doi:10.1002/fsn3.1492)
Supplement: Supplementary file 19 — Table S5 [file FSN3-8-1766-s019.docx]

**Supplementary table 5. Characteristics of studies reporting the effect of coenzyme Q10 (CoQ10) on catalase (CAT) included in the systematic review.**

| **Study** | **Study design** | **Population** | **Intervention** | **Duration** | **CoQ10 group** | | **Placebo group** | | **P-value**  **(Between group)** | **Main**  **outcomes** |
| --- | --- | --- | --- | --- | --- | --- | --- | --- | --- | --- |
|  |  |  |  |  | **^1^B** | **^2^A** | **^1^B** | **^2^A** |  |  |
| Lee  et al (2012) | Randomized double-blind, placebo- controlled trial, parallel | CAD patients  (Total n =32; Completed study: intervention: 15, placebo: 12) | CoQ10  (150 mg/d) or placebo | 84 days | 42±37 | 45.81± 60.22 | 65.56±34.76 | 13.88± 3.86 | **Between groups:**  0.03 | BUN, Cr, TC, TG, LDL, HDL, hs-CRP, IL-6, COQ10, MDA, SOD, homocysteine, CAT |
| Lee  et al (2012) | Randomized double-blind, placebo- controlled trial, parallel | CAD patients  (Total n=32; Completed study: intervention: 16, placebo: 12) | CoQ10  (60 mg/d) or placebo | 84 days | 50± 48 | 23± 15 | 65.56±34.76 | 13.88± 3.86 | **Between groups:**  p<0.05 | BUN, Cr, TC, TG, LDL, HDL, hs-CRP, IL-6, COQ10, MDA, SOD, homocysteine, CAT |
| Lee  et al (2013) | Randomized double-blind, placebo- controlled trial, parallel | CAD patients  ( Total n=51; Completed study: intervention: 23, placebo: 19) | CoQ10  (300 mg/d) or placebo | 84 days | 21± 4 | 26± 11 | 24± 6 | 22 ± 6 | **Between groups**:  0.033 | Cr, TC, TG, LDL, HDL, hs-CRP, TNF-α, IL-6, adiponectin, COQ10, Vit E, SOD, GPx, CAT |
| Liu  et al (2016) | Randomized double-blind, placebo- controlled trial, parallel | HCC patients  (Total n= 41; Completed study: intervention: 20, placebo: 19) | CoQ10  (300 mg/d) or placebo | 84 days | 14.74± 7.63 | 18.00±6.82 | 16.36± 7.97 | 15.24 ± 6.62 | **Between groups**:  0.01 | COQ10, Vit E, hs-CRP, IL-6, BUN, Cr, GOT, GPT, TC, TG, LDL, HDL, TC / HDL, TNF-α, MDA, SOD, CAT, GPx |
| Yen  et al (2018) | Randomized double-blind, placebo- controlled trial, parallel | T2DM patients  (Total n =50; Completed study: intervention: 24, placebo: 23) | liquid ubiquinol  (100 mg/d) or placebo | 84 days | 13.56± 4.96 | 16.27± 5.21 | 14.46± 4.97 | 17.67± 7.89 | **Between groups:**  p<0.05 | COQ10, glucose homoeostasis parameters, lipid profiles, oxidative stress and anti-oxidative enzyme activities |
| Zarei  et al (2018) | Randomized double-blind, placebo- controlled trial, parallel | T2DM patients  (Total n =68; Completed study: intervention: 34, placebo: 34) | CoQ10  (100 mg/d) or placebo | 84 days | 2.08±0.15 | 3.48±0.19 | 2.17±0.09 | 2.44±0.08 | **Between groups:**  0.05 | FBS, HgA1C, insulin, QUICKI, CoQ10, α‑amylase, ADA, CAT, TAC |

^1^B: Before intervention; ^2^A: After intervention. CoQ10: Coenzyme Q10; CAD: coronary artery disease; BUN: blood urea nitrogen; Cr: creatinine; TC: Total Cholesterol; TG: Triglyceride; LDL: Low Density Lipoprotein; HDL: High Density Lipoprotein; hs-CRP: High Sensitivity C-reactive Protein; IL-6: Interleukin 6; MDA: Malondialdehyde; SOD: Superoxide Dismutase; TNF-α: Tumor Necrosis Alpha; GPx: Glutathione Peroxidase; CAT: Catalase; HCC: hepatocellular carcinoma; GOT: glutamic oxaloacetic transaminase; GPT: glutamic pyruvic transaminase; T2DM: Type 2 Diabetes Mellitus; FBS: Fasting blood sugar; HgA1C: Hemoglobin A1C; QUICKI: Quantitative insulin sensitivity check index; ADA: Adenosine deaminase; TAC: Total antioxidant capacity. All values have been presented as mean±SD.
