# Supplementary material for: The effect of coenzyme Q10 supplementation on oxidative stress: A systematic review and meta‐analysis of randomized controlled clinical trials
Source: Food Sci Nutr. 2020 Mar 19;8(4):1766–76. doi: 10.1002/fsn3.1492 (PMC7174219; doi:10.1002/fsn3.1492)
Supplement: Supplementary file 20 — Table S6 [file FSN3-8-1766-s020.docx]

**Supplementary table 6. Characteristics of studies reporting the effect of coenzyme Q10 (CoQ10) on Isoprostanes included in the systematic review.**

| **Study** | **Study design** | **Population** | **Intervention** | **Duration** | **CoQ10 group** | | **Placebo group** | | **P-value**  **(Between groups)** | **Main**  **outcomes** |
| --- | --- | --- | --- | --- | --- | --- | --- | --- | --- | --- |
|  |  |  |  |  | **^1^B** | **^2^A** | **^1^B** | **^2^A** |  |  |
| Dai  et al (2011) | Randomized double-blind, placebo- controlled trial, parallel | ischemic LVSD patients (Total n=56; Completed study: intervention: 28; placebo: 28) | CoQ10  (300 mg/d) or placebo | 56 days | 525± 397 | Not reported | 508±319 | Not reported | **Before intervention:**  0.86  **After intervention:**  0.09 | FBS, HbA1c, TG, LDL, HDL, TC, hs-CRP, SBP, DBP, Cr, ApoA-I, ApoB, LPa, lactate, pyruvate, SOD, 8-isoprostane, FMD |
| **^¶^**Rivara  et al (2016) | Randomized double-blind, placebo- controlled trial, parallel | Hemodialysis patients  (Total n=54; Completed study: intervention: 16; placebo: 23) | CoQ10  (600 mg/d) or placebo | 120 days | 45.6  (35.1-56.1) | 39.2  (30.9-47.6) | 49.3  (40.6-58.0) | 45.1  (36.6-53.6) | **Between groups:**  0.3 | Total CoQ10, F2-isoprostanes, sofurans, TnT, NT-proBNP, SBP, DBP |
| **^¶^**Rivara  et al (2016) | Randomized double-blind, placebo- controlled trial, parallel | Hemodialysis patents  (Total n=54; Completed study: intervention: 19; placebo: 23) | CoQ10  (1200 mg/d) or placebo | 120 days | 62.4  (43.5-81.3) | 47.5  (33.2-61.8) | 49.3  (40.6-58.0) | 45.1  (36.6-53.6) | **Between groups:**  0.002 | Total CoQ10, F2-isoprostanes, sofurans, TnT, NT-proBNP, SBP, DBP |
| Gholami  et al (2018) | Randomized double-blind, placebo- controlled trial, parallel | T2DM patients  (Total n= 68; Completed study: intervention: 34; placebo: 34) | CoQ10  (100 mg/d) or placebo | 84 days | 407.40 ±16.50 | 338.22± 15.54 | 379.91 ±14.47 | 375.41 ±15.76 | **Before intervention:**  0.215  **After intervention:**  0.098 | FBG, HbA1C, Insulin, TC, TG, HDL, LDL, CoQ10, CoQ10/TC, APN, Leptin, MDA, 8-Isoprostane, HOMA-IR |
| Hamilton  et al (2008) | Randomized double-blind, placebo- controlled trial, crossover | T2DM patients  (Total n=23) | CoQ10(200 mg/d) or placebo | 84 days | 1.284 ±70 | 1.298 ±69 | 1.302 ±68 | 1.275 ±86 | **Between groups:**  0.58 | CoQ10, F2-isoprostanes, 20-HETE, GHb, LDL, Brachial artery FMD & NMD |

^1^B: Before intervention; ^2^A: After intervention. CoQ10: Coenzyme; LVSD: left ventricular ejection fraction; FBS: Fasting Blood Sugar; HbA1C: [Hemoglobin A1c; TG: Triglyceride; LDL: Low Density Lipoprotein; HDL: High Density Lipoprotein; TC: Total Cholesterol; hs-CRP: High Sensitivity C-reactive Protein; SBP: Systolic Blood Pressure; DBP: Diastolic Blood Pressure; Cr: creatinine; ApoA-I:](https://www.google.com/url?sa=t&rct=j&q=&esrc=s&source=web&cd=1&cad=rja&uact=8&ved=2ahUKEwiRhprmkt_gAhVD16QKHUxlAuwQFjAAegQIChAB&url=https%3A%2F%2Fwww.webmd.com%2Fdiabetes%2Fguide%2Fglycated-hemoglobin-test-hba1c&usg=AOvVaw1b3BeTdIzX-FVOlrwKTuAz) [Apolipoprotein A1; Apo-B:](https://www.google.com/url?sa=t&rct=j&q=&esrc=s&source=web&cd=1&cad=rja&uact=8&ved=2ahUKEwjsyJqHkd_gAhXSKlAKHXH2DtYQFjAAegQICRAB&url=https%3A%2F%2Fen.wikipedia.org%2Fwiki%2FApolipoprotein_A1&usg=AOvVaw0-jQju5nOcIQoWw9jh84TB) [Apolipoprotein B; LPa: lipoprotein a; SOD: Superoxide Dismutase; FMD : flow-mediated dilatation; TnT: Troponin T; NT-proBNT: N- terminal-pro-brain natriuretic peptide;T2DM: Type 2 Diabetes Mellitus; FPG: Fasting Plasma Glucose;](https://www.google.com/url?sa=t&rct=j&q=&esrc=s&source=web&cd=1&cad=rja&uact=8&ved=2ahUKEwiE4ra7kd_gAhVDZlAKHem6CrkQFjAAegQICRAB&url=https%3A%2F%2Fen.wikipedia.org%2Fwiki%2FApolipoprotein_B&usg=AOvVaw30liehWrD-pNz6lHuI41wW)  [APN:Adiponectin; MDA: Malondialdehyde; HOMA-IR: Homeostasis Model Assessment-Insulin Resistance; CAT: Catalase; SOD: Superoxide Dismutase;20-HETH: Hydroxieicosatetraenoic acid; GHb: Gama hydroxybutyrate; NMD: Nitroglycerin-mediated vasodilations. All values have been presented as mean±SD except studies of ¶Rivara et al (2016) that their values presented as squares means (95% confidence interval).](https://www.google.com/url?sa=t&rct=j&q=&esrc=s&source=web&cd=1&cad=rja&uact=8&ved=2ahUKEwiRhprmkt_gAhVD16QKHUxlAuwQFjAAegQIChAB&url=https%3A%2F%2Fwww.webmd.com%2Fdiabetes%2Fguide%2Fglycated-hemoglobin-test-hba1c&usg=AOvVaw1b3BeTdIzX-FVOlrwKTuAz)
