# Supplementary material for: The effect of coenzyme Q10 supplementation on oxidative stress: A systematic review and meta‐analysis of randomized controlled clinical trials
Source: Food Sci Nutr. 2020 Mar 19;8(4):1766–76. doi: 10.1002/fsn3.1492 (PMC7174219; doi:10.1002/fsn3.1492)
Supplement: Supplementary file 21 — Table S7 [file FSN3-8-1766-s021.docx]

**Supplementary table 7. Characteristics of studies reporting the effect of coenzyme Q10 (CoQ10) on Glutathione (GSH) included in the systematic review.**

| **Study** | **Study design** | **Study population** | **intervention** | **Duration** | **CoQ10 group** | | **Placebo group** | | **P-value**  **(Between group)** | **Main**  **outcomes** |
| --- | --- | --- | --- | --- | --- | --- | --- | --- | --- | --- |
|  |  |  |  |  | **^1^B** | **^2^A** | **^1^B** | **^2^A** |  |  |
| Raygan  et al (2016) | Randomized double-blind, placebo- controlled trial, parallel | T2DM patients with CHD  (Total n= 60; Completed study: intervention: 30; placebo: 30) | CoQ10  (100 mg/d) or placebo | 56 days | 0.34 ±0.1 | 0.41 ±0.09 | 0.52 ±0.52 | 0.49 ±0.16 | **Between groups:**  0.06 | FPG, Insulin, HOMA-IR, TC, HOMA-B, QUICKI, TG, VLDL, LDL, HDL, hs-CRP, NO, TAC, GSH, MDA |
| Fallah  et al (2019) | Randomized double-blind, placebo- controlled trial, | T2DM patients with HD  (Total n=60; Completed study: intervention: 30; placebo: 30) | CoQ10  (120 mg/d) or placebo | 84 days | 415.155±58.467 | 420.82±67.762 | 520.653±0.212 | 5.02±3.42  (µmol/L) | **Between groups:**  0.053 | TAC, GSH, MDA, hs-CRP,NO |

^1^B: Before intervention; ^2^A: After intervention. CoQ10: Coenzyme Q10; T2DM: Type 2 Diabetes Mellitus; FPG: Fasting Plasma Glucose; HOMA-IR: Homeostasis Model Assessment-Insulin Resistance; TC: Total Cholesterol; HOMA-B: homeostasis model assessment of f β-cell function; QUICKI: Quantitative Insulin Sensitivity Check Index; TG: Triglyceride; VLDL: Very Low Density Lipoprotein; LDL: Low Density Lipoprotein; HDL: High Density Lipoprotein; hs-CRP: High Sensitivity C-reactive Protein; NO: Nitric Oxide; TAC: Total Antioxidant Capacity; GSH: [Glutathione; MDA: Malondialdehyde. All values have been presented as mean±SD..](https://www.google.com/url?sa=t&rct=j&q=&esrc=s&source=web&cd=1&cad=rja&uact=8&ved=2ahUKEwj2n6j3k9_gAhUC3aQKHZZ8CwcQFjAAegQICxAB&url=https%3A%2F%2Fen.wikipedia.org%2Fwiki%2FGlutathione&usg=AOvVaw25nLLayE-z9n4Ti_v3ZJlv)
