# Supplementary material for: The effect of coenzyme Q10 supplementation on oxidative stress: A systematic review and meta‐analysis of randomized controlled clinical trials
Source: Food Sci Nutr. 2020 Mar 19;8(4):1766–76. doi: 10.1002/fsn3.1492 (PMC7174219; doi:10.1002/fsn3.1492)
Supplement: Supplementary file 22 [file FSN3-8-1766-s022.docx]

**Supplementary figures legends**

**Supplementary figure 1.** Sensitivity analysis was performed using a random-effect model for impact of coenzyme Q10 (CoQ10) on total antioxidant capacity (TAC) concentration.

**Supplementary figure 2.** Forest plot illustrates weighted mean difference (represented by the black square) and 95 % confidence interval (CI) (represented by horizontal line) for total antioxidant capacity (TAC) level and coenzyme Q10 (CoQ10) in different doses (A. dose= 100 or > 100 mg/d) and intervention durations (B. duration ≤ 60 or > 60 days). Weights are from random effects analysis. The area of the black square is proportional to the specific study weight to the overall meta-analysis. The center of the diamond displays the pool weighted mean difference and its width shows the pooled 95 % CI. Std diff, standard difference.

**Supplementary figure 3.** Sensitivity analysis was performed using a random-effect model for impact of coenzyme Q10 (CoQ10) on malondialdehyde (MDA) level.

**Supplementary figure 4.** Forest plot illustrates weighted mean difference (represented by the black square) and 95 % confidence interval (CI) (represented by horizontal line) for malondialdehyde (MDA) level and coenzyme Q10 (CoQ10) in the different doses (A. dose ≤ 100 or > 100 mg/d), intervention durations (B. duration ≤ 60 or > 60 days) and studies with different qualities (C. high quality or low quality). Weights are from random effects analysis. The area of the black square is proportional to the specific study weight to the overall meta-analysis. The center of the diamond displays the pool weighted mean difference and its width shows the pooled 95 % CI. Std diff, standard difference.

**Supplementary figure 5.** Sensitivity analysis was performed using a random-effect model for impact of coenzyme Q10 (CoQ10) on glutathione peroxidase (GPx) level.

**Supplementary figure 6.** Forest plot illustrates weighted mean difference (represented by the black square) and 95 % confidence interval (CI) (represented by horizontal line) for glutathione peroxidase (GPx) level and coenzyme Q10 (CoQ10) in the different doses (A. dose ≤ 100 or > 100 mg/d) and studies with different qualities (B. high quality or low quality). Weights are from random effects analysis. The area of the black square is proportional to the specific study weight to the overall meta-analysis. The center of the diamond displays the pool weighted mean difference and its width shows the pooled 95 % CI. Std diff, standard difference.

**Supplementary figure 7.** Sensitivity analysis was performed using a random-effect model for impact of coenzyme Q10 (CoQ10) on superoxide dismutase (SOD) level.

**Supplementary figure 8.** Forest plot illustrates weighted mean difference (represented by the black square) and 95 % confidence interval (CI) (represented by horizontal line) for superoxide dismutase (SOD) level and coenzyme Q10 (CoQ10) in the different doses (A. dose ≤ 100 or > 100 mg/d) and studies with different qualities (B. high quality or low quality). Weights are from random effects analysis. The area of the black square is proportional to the specific study weight to the overall meta-analysis. The center of the diamond displays the pool weighted mean difference and its width shows the pooled 95 % CI. Std diff, standard difference.

**Supplementary figure 9.** Sensitivity analysis was performed using a random-effect model for impact of coenzyme Q10 (CoQ10) on catalase (CAT) level.

**Supplementary figure 10.** Forest plot illustrates weighted mean difference (represented by the black square) and 95 % confidence interval (CI) (represented by horizontal line) for catalse (CAT) level and coenzyme Q10 (CoQ10) in the different doses (A. dose ≤ 100 or > 100 mg/d) and studies with different qualities (B. high quality or low quality). Weights are from random effects analysis. The area of the black square is proportional to the specific study weight to the overall meta-analysis. The center of the diamond displays the pool weighted mean difference and its width shows the pooled 95 % CI. Std diff, standard difference.

**Supplementary Figure 11.** Meta-regression plots of the association between Standardized mean difference in oxidative stress markers levels values (A: total antioxidant capacity, B: malondialdehyde, C: glutathione peroxidase, D: superoxidase dismutase, E: catalase) after coenzyme Q10 (CoQ10) supplementation with dose of CoQ10. The size of each circle is inversely proportional to the variance of change.

**Supplementary Figure 12.** Meta-regression plots of the association between Standardized mean difference in oxidative stress markers levels values (A: malondialdehyde, B: total antioxidant capacity) after coenzyme Q10 (CoQ10) supplementation with duration of trial. The size of each circle is inversely proportional to the variance of change.

**Supplementary Figure 13.** Funnel plot (with 95 % confidence intervals) of Standardized mean differences for oxidative stress markers (A: total antioxidant capacity, B: malondialdehyde, C: glutathione peroxidase, D: superoxidase dismutase, E: catalase) concentrations with coenzyme Q10 (CoQ10). The vertical line (——) shows the combined mean differences calculated with the random-effects model.

**Supplementary Figure 14.** Trim and fill method was used to impute for potentially missing studies for superoxidase dismutase (A) and catalase (B), open circles represent observed published studies; open diamond represents observed effect size; closed diamond represents imputed effect size; closed circles represents imputed studies.
